# Novel subtypes of metabolic associated steatotic liver disease linked to clinical outcomes: implications for precision medicine

**DOI:** 10.1186/s12967-025-06670-5

**Published:** 2025-07-10

**Authors:** Chang Hong, Sheng-xing Liang, Ze-yang Li, Rui-ning Li, Hong-bo Zhu, Mingfei He, Hao Cui, Jing-zhe He, Yan Li, Jia-ren Wang, Xue-jing Zou, Wen-yuan Li, Lin Zeng, Li Liu, Lu-shan Xiao

**Affiliations:** 1 Department of Health Management, Nanfang Hospital, Southern Medical University, Guangzhou, 510515 China; 2 Guangdong Provincial Key Laboratory of Viral Hepatitis Research, Department of Infectious Diseases, Nanfang Hospital, Southern Medical University, Guangzhou, 510515 China; 3 School of Public Health, Southern Medical University, Guangzhou, 510515 China; 4 Nanfang Hospital, Southern Medical University, Guangzhou, 510515 China; 5 School of Health Management, Southern Medical University, Guangzhou, 510515 China; 6 Department of Oncology, The First Affiliated Hospital, Hengyang Medical School, University of South China, Hengyang, 421001 Hunan China

**Keywords:** MASLD, Cluster, Clinical indicator, Complications, Genome-wide association study (GWAS)

## Abstract

**Background & aims:**

Although metabolic dysfunction-associated steatotic liver disease (MASLD) is associated with high multimorbidity and mortality, existing classification systems and risk prediction models largely ignore the heterogeneity of MASLD. Improved subtype definition could improve prediction of outcomes and inform new precision treatment strategies.

**Methods:**

We analyzed individuals with MASLD from population-based electronic health record resource from UK Biobank (n = 125,197) and Health examinee dataset of Nanfang Hospital (n = 995). We identified subtypes with K-means clustering method. The Cox proportional hazard regression model analyzed the relationship between variables and MASLD-related complications.

**Results:**

After identifying five clusters across seven clinical indicators which were age, body mass index, monocyte/lymphocyte ratio, aspartate aminotransferase, waist-hip ratio, low-density lipoprotein-cholesterol, and cholesterol, we labelled MASLD subtypes: (1) Metabolic-Dyslipidemia, (2) Younger, (3) Obesity, (4) Inflammatory, and (5) Hepatotoxic. Metabolic outcomes differed across these five subtypes. Hepatotoxic MASLD showed an increased risk of severe liver diseases compared to Metabolic-Dyslipidemia MASLD [HR = 13.9, 95% CI 10.7–18.1]. The extrahepatic complications were highest in Inflammatory MASLD. These two groups were defined as the high-risk group with higher health burden than other three groups, which classified as low-risk group. Differential single-nucleotide polymorphisms were concentrated on chromosomes 1 and 19 when comparing the high- and low-risk groups, and the annotated genes enrichment pathways were primarily related to lipid metabolism and transport.

**Conclusions:**

Patient subtypes derived by clinical indicators are a valuable addition to existing MASLD classification systems, which could provide a valuable tool to aid in selecting specific treatment approaches.

**Graphical Abstract:**

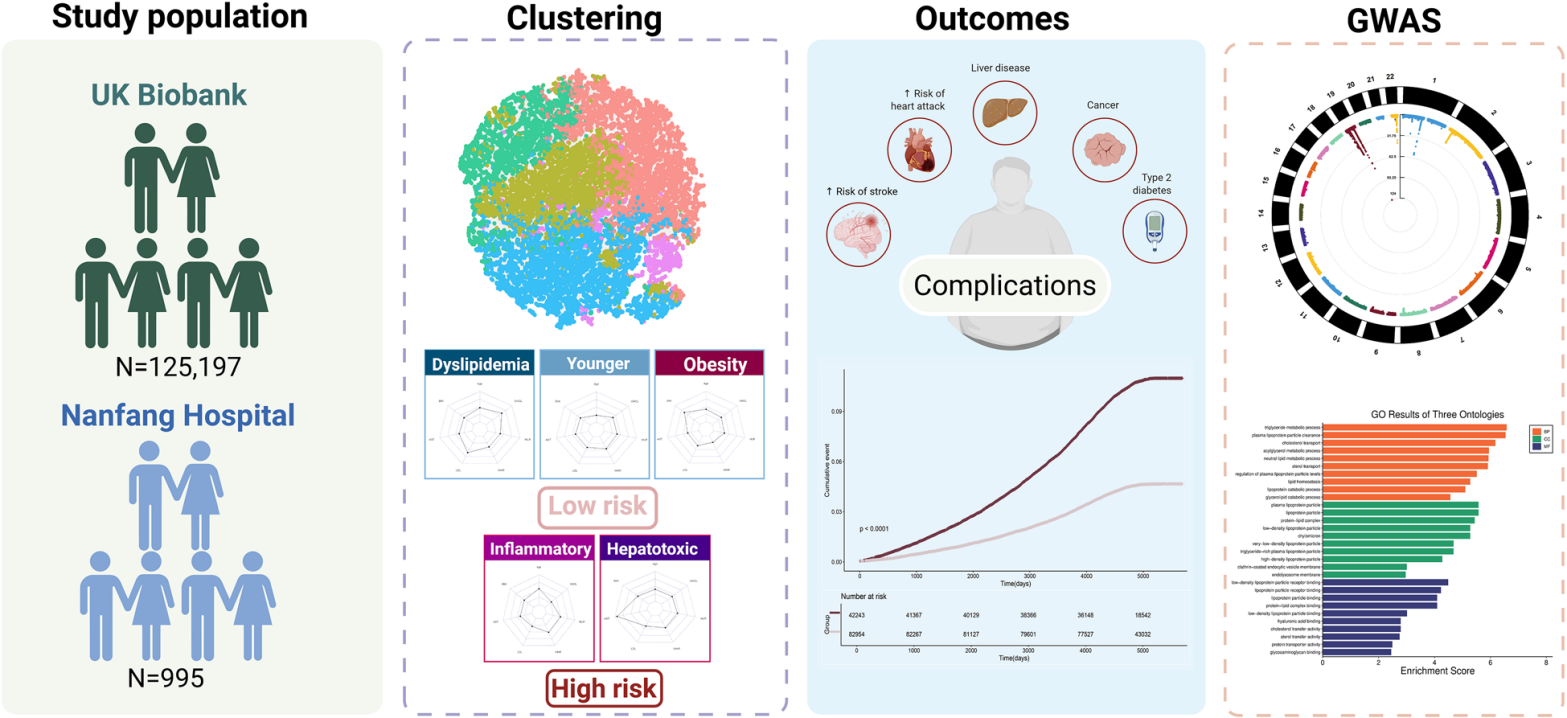

**Supplementary Information:**

The online version contains supplementary material available at 10.1186/s12967-025-06670-5.

## Introduction

Metabolic dysfunction-associated steatotic liver disease (MASLD) is an increasingly prevalent chronic liver disease, affecting 20–30% of the global population [1–3]. MASLD is a multisystem disease driven by systemic insulin resistance and metabolic dysfunction [2]. These factors contribute not only to liver-related complications (cirrhosis, liver failure, and hepatocellular carcinoma) [1, 4, 5] but also to extrahepatic manifestations, including cardiovascular disease, type 2 diabetes mellitus, and chronic kidney disease [6–10]. Moreover, population-based follow-up studies into the causes of death for individuals with MASLD have indicated that approximately half of the deaths are related to complications, like atherosclerosis [11]. Heterogeneity is an essential feature in the development of MASLD, while the current severity stratification of MASLD is a simple dichotomy (i.e., non-steatohepatitis and steatohepatitis) [12] and does not distinguish between different MASLD types. Given the increasing rates of multimorbidity in individuals with MASLD, a refined classification that can identify patients at high risk of complications is necessary for new aetiologic insights and potential therapy for MASLD.

The widespread implementation of electronic health records and machine learning enhances disease subtyping and risk stratification, facilitating early intervention and precision medicine approaches [13, 14]. Specifically, unsupervised machine learning, a data mining method aims to detect unknown patterns in data without the need for prior human knowledge and intervention [15]. As an unsupervised machine learning algorithm, K-means clustering offers valuable applications in clinical research and practice. It can function as a dimensionality reduction technique, where the derived clusters may then be incorporated as variables in conventional regression analyses [16–18].

To better identify subtypes and perform risk prediction of MASLD, we conducted a cluster analysis based on clinical indicators related to the occurrence and development of MASLD and examine the association between these subgroups and the risk of intrahepatic and extrahepatic complications associated with MASLD, ultimately aiming to provide insights for more precise treatment approaches tailored to individual patients.

## Materials and methods

### Study population

**Fig. 1:**
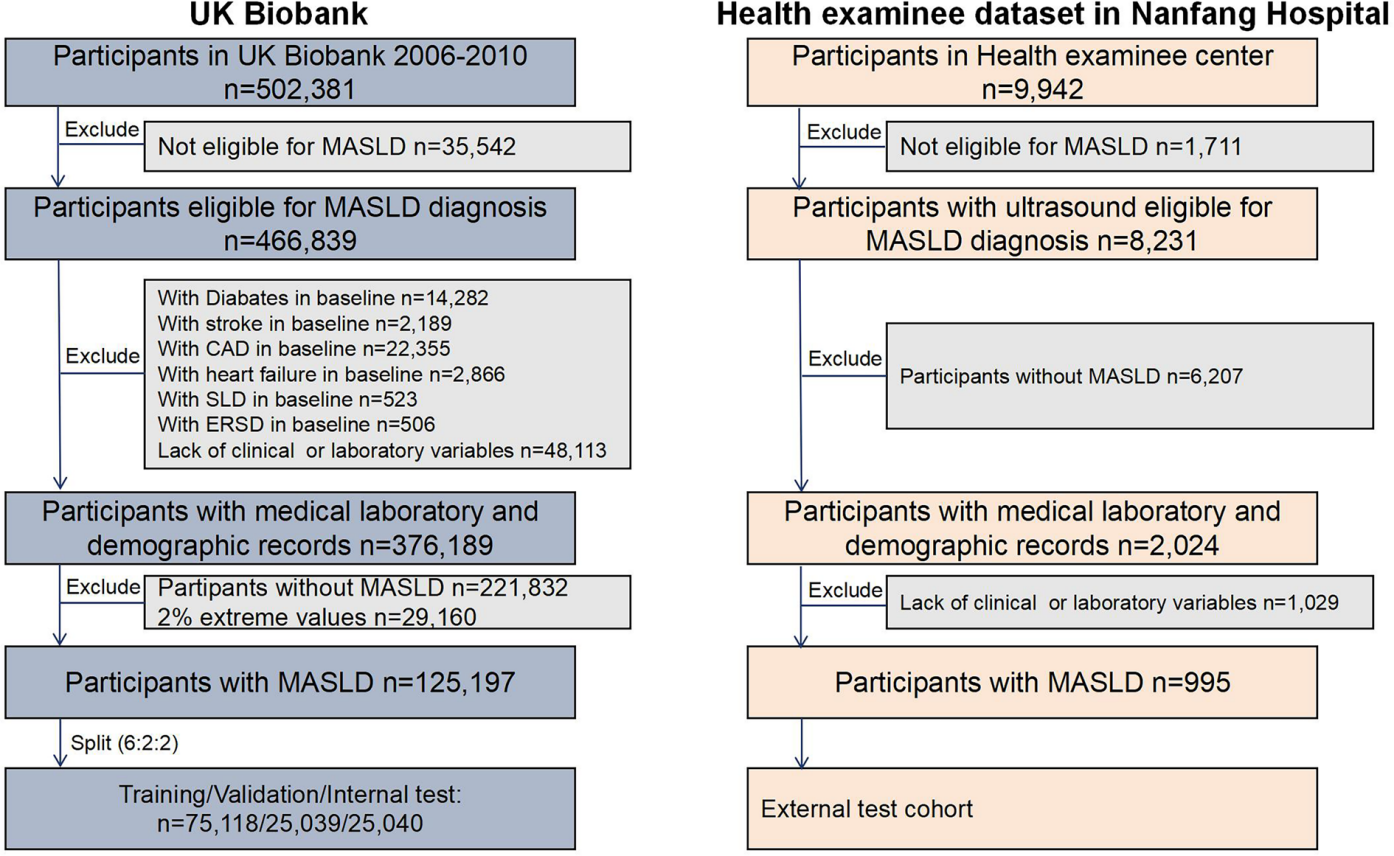
The flowchart of study participants

The UK Biobank is a cohort study that enrolled over 500,000 participants aged 40–69 years at baseline [19] between 2006 and 2010. After excluding subjects, a total study population of 502,381 was obtained. Participants were excluded from MASLD diagnosis if they had missing data for any of the components of fatty liver index (BMI, waist circumference, triglycerides, or gamma-glutamyl transferase), as this precluded the calculation of FLI and thus the assessment of hepatic steatosis. And 466,839 participants were eligible for MASLD diagnosis in UK Biobank. The final analysis included 125,197 participants with complete serological indicators (Supplemental Table 1), age, sex, and other characteristics (Fig. 1 and Supplemental method 1).

The health examinee dataset of Nanfang Hospital, designed as the external test cohort in the study, contains records on population undergoing health checkups. We extracted data from individuals aged ≥ 18 years who visited hospital between 2015 and 2020 continuously (n = 9942). The flowchart used to determine the specific inclusion and exclusion criteria is shown in Fig. 1 and the workflow is shown in Supplemental Fig. 1.

### Definition of MASLD, MASLD-related fibrosis, and complications

The fatty liver index, encompassing triglycerides, body mass index (BMI), gamma-glutamyl transferase, and waist circumference, was utilized to predict steatotic liver disease. A score of ≥ 60 on this index indicated a steatotic liver [20–22]. MASLD was diagnosed based on the fatty liver index in UK Biobank or abdominal ultrasonography in Health examinee dataset of Nanfang Hospital, in addition to presence of at least one of five cardiometabolic risk factors [1, 23, 24]. 

Fibrosis-4 (FIB-4) was chosen as the diagnostic panel to identify participants with advanced fibrosis as it is well-validated and supported by the literature [25, 26]. FIB-4 levels were calculated based on age, alanine aminotransferase and aspartate aminotransferase levels, and platelet counts.

Incident SLD was defined as a composite diagnosis of cirrhosis, decompensated liver disease (portal hypertension, esophageal varices with or without bleeding, hepatorenal syndrome, and liver failure), hepatocellular carcinoma, or liver transplantation (ICD-10 C22.0, I85.0, I85.9, K70.3, K70.4, K72.1, K72.9, K74.1, K74.2, K74.6, K76.6, K76.7, and Z94.4) in any of the aforementioned records [27].

Stroke was defined according to the ICD-10 as follows: stroke (I60, I61, I62.9, I63, I64, I67.8, I69.0, and I69.3), ischemic stroke (I63), and hemorrhagic stroke (I60 and I62.9) [28]. Incident HF was defined as hospital admission according to the ICD-10 codes I11.0, I13.0, I13.2, I50.0, I50.1, and I50.9 [29].

Incident ESRD was defined when patients commenced treatment with renal replacement therapy [30]. ESRD was identified in the hospital admission data using ICD-10 and OPCS4 codes. Participants who underwent kidney transplantation or peritoneal dialysis were assumed to have ESRD. 

The follow-up time for each participant was calculated from the baseline until the date of identification of any outcome, loss to follow-up, or last follow-up (2022-11-14), whichever occurred first.

### Feature selection and K-means clusters

This study used the correlation coefficient method to screen clustering features. To calculate correlation coefficient scores, a supervised learning algorithm was primarily utilized for generating continuous output values. The rank correlation coefficient between the selected continuous features and the noninvasive serological index FIB-4 score was calculated to screen for relevant features using Spearman’s rank correlation coefficient (ρ). For cluster analysis, clustering features were selected based on the criterion of high correlation with the FIB-4 scores, which were age, aspartate aminotransferase (AST), waist-hip ratio (WHR), monocyte/lymphocyte ratio (MLR), low-density lipoprotein-cholesterol (LDL-C), cholesterol (CHOL), and BMI (shown in Supplemental Fig. 2 A). 

In this study, the unsupervised clustering method, K-means clustering, was employed. The selection basis for determining the number of clusters by integrating the DB index and the elbow plot (Supplemental Fig. 2B). We used it to identify clusters with acceptable stability in the training group. Subsequently, we examined cluster distribution in the internal validation and test sets and external test sets, aiming to achieve a similar distribution as was observed in the training set.

### Genome-wide association study (GWAS) and pathway enrichment analysis

A GWAS was conducted to identify genetic variation polymorphisms across individuals’ entire genome. The UK Biobank has conducted genome-wide genotyping sequencing for participants [31]. Genotyping was performed using the Affymetrix UK BiLEVE Array and the UK Biobank Axiom Array. For the cross-trait GWAS 118, 282 subjects of European ancestry from the UK Biobank were included. Exclusion criteria included: (1) Incomplete genotyping or low call rate (< 98%) or incorrect gender information, n = 2421; (2) Restricted to genetically white British ancestry or Removed outliers based on principal component analysis of genetic ancestry, n = 3724; (3) Technical Artifacts: Excessive heterozygosity or High genotype missingness (> 5%), n = 770. These selected single-nucleotide polymorphism (SNP) sites were used for GWAS analysis.

The study identified five clusters based on severe complications, dividing them into high-risk and low-risk groups. Generalized linear mixed model (GLMM)-based genome-wide association (fastGWA-GLMM) analyses were performed [32], adjusting for age, sex, genotyping array, and the top 20 ancestry principal components. The ability to detect independent association signals was compared, and functional annotation and enrichment analyses were conducted for the associated loci.

### Statistical analysis

Continuous variable distributions were expressed as a median and interquartile range, and categorical variables were expressed as frequencies and percentages. The chi-square and Kruskal–Wallis rank sum tests were used to evaluate the intergroup equilibrium of different characteristics. The Kaplan–Meier method was used to estimate the prognosis of the subgroups, and the logarithmic rank test was used to compare the differences among different subtypes. Cox regression models were used to estimate hazard ratios (HR) and 95% confidence intervals (CI) for different outcomes across clusters. All statistical analyses were performed using R version 4.2.2. All tests were two-sided, and factors with *P* values ≤ 0·05 were considered statistically significant.

## Results

### Characteristics of patients in different datasets

This study comprised 125,197 and 995 participants diagnosed with MASLD whose data were sourced from the UK Biobank and Nanfang Hospital. The average age of participants in the UK Biobank cohort was greater than that of participants in the Nanfang Hospital cohort (58 vs. 46 years; *P* < 0.001), because the participants in the health examinee dataset were largely working-age population whose overall age was young. Participants from UK Biobank had a higher BMI and higher TG, CHOL, LDL-C, AST, and ALT levels (*P* < 0.001; Table 1). The cohort consisted of 78,791 men (mean age: 58 years) and 46,406 women (mean age: 59 years), with a majority of them being white individuals (92%, as shown in Table 1). Participants in UK Biobank were randomly assigned to the training, validation, and test cohorts in a ratio of 6:2:2 (Supplemental Table 2).

**Table 1:** Baseline characteristic of MASLD population

Characteristic	UK Biobank n = 125,197	Nanfang Hospital n = 995	*P* value^1^	Female n = 46,406	Male n = 78,791	*P* value^2^
Age	58 (51, 63)	46 (38, 52)	< 0.001	59 (52, 63)	58 (50, 63)	< 0.001
Sex	78,791(63%)	627 (63%)	0.75	–	–	–
Race		–				0.8
White	114,904 (92%)	–		42,600 (91.8%)	72,304 (91.8%)	
Else	10,293 (8.2%)	–		3806 (8.2%)	6487 (8.2%)	
BMI	30.5 (28.3, 33.3)	25.39 (23.59, 27.27)	< 0.001	32.4 (29.9, 35.5)	29.5 (27.7, 31.8)	< 0.001
WHR	0.93 (0.88, 0.98)	0.89 (0.83, 0.94)	< 0.001	0.87 (0.83, 0.91)	0.96 (0.93, 1.00)	< 0.001
ALT	26 (20, 35)	22 (16, 32)	< 0.001	22 (17, 30)	28 (22, 37)	< 0.001
AST	26 (23, 31)	20 (16, 24)	< 0.001	24 (21, 29)	27 (24, 32)	< 0.001
ALB	45.18 (43.46, 46.90)	45.10 (43.40, 46.50)	0.01	44.45 (42.80, 46.14)	45.60 (43.92, 47.27)	< 0.001
TG	2.14 (1.58, 2.92)	1.64 (1.20, 2.40)	< 0.001	2.00 (1.49, 2.69)	2.23 (1.65, 3.04)	< 0.001
CHOL	5.85 (5.11, 6.62)	5.41 (4.73, 6.10)	< 0.001	6.06 (5.29, 6.85)	5.74 (5.01, 6.48)	< 0.001
HDL-C	1.23 (1.07, 1.44)	1.26 (1.10, 1.43)	< 0.001	1.36 (1.18, 1.56)	1.17 (1.02, 1.34)	< 0.001
LDL-C	3.75 (3.18, 4.34)	3.38 (2.92, 3.90)	< 0.001	3.85 (3.26, 4.46)	3.69 (3.13, 4.26)	< 0.001
HbA1c	5.43 (5.20, 5.67)	–		5.48 (5.26, 5.73)	5.40 (5.17, 5.64)	< 0.001
MLR	0.25 (0.19, 0.31)	0.18 (0.15, 0.22)	< 0.001	0.21 (0.17, 0.27)	0.27 (0.21, 0.34)	< 0.001

n (%); Median (IQR); P value, Kruskal–Wallis rank sum test; Pearson’s Chi-squared test

*BMI* body mass index, *WHR* waist-hip ratio, *ALT* alanine aminotransferase, *AST* aspartate aminotransferase, *TG* triglyceride, *CHOL* cholesterol, *HDL-C* high-density lipoprotein cholesterol, *LDL-C* low-density lipoprotein cholesterol, *HbA1c* Hemoglobin A1c, *MLR* monocyte/lymphocyte ratio

^1^ UK Biobank vs. Nanfang Hospital

^2^ Female vs. Male in UK Biobank

### Cluster analysis of the MASLD cohort

**Fig. 2:**
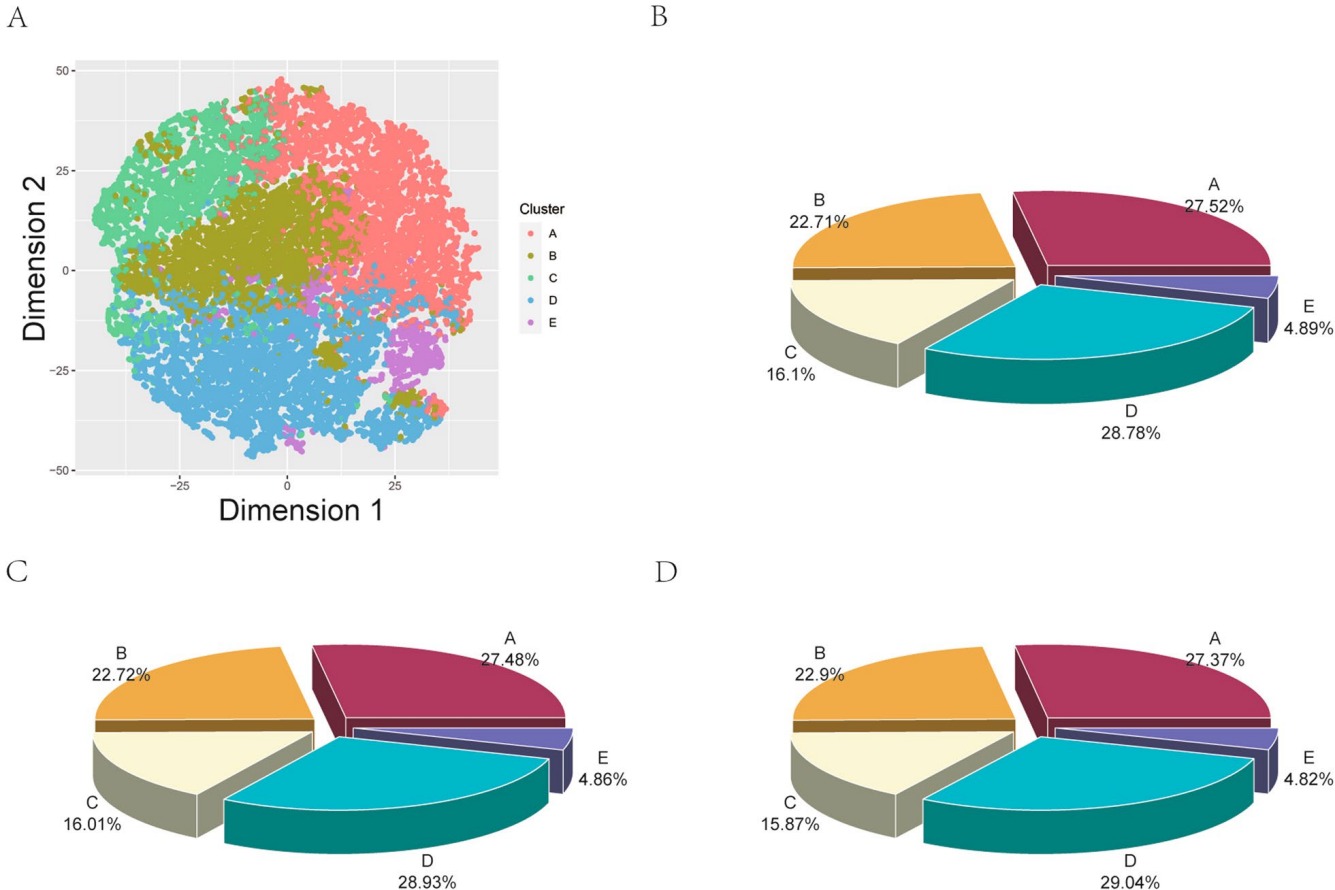
Visualization of the clustering and patient distribution according to method of classification. **A** t-SNE visualization of training group in the two-dimensional space, metric = Euclidean. All data points are labelled with k-means clustering. Distribution of patients in the training cohort (**B**), validation cohort (**C**) and internal test cohort (**D**)

Five distinct clusters were identified through cluster analysis of the training group (Fig. 2A). The distribution of each cluster across the training, validation, and test cohorts is shown in Fig. 2B–D. The baseline characteristics of the training cohort are shown in Table 2 and Supplemental Fig. 3. Cluster A, labelled as Metabolic-Dyslipidemia MASLD, included 20,682 (27.5%) of the 75,118 participants in the training cohort and was characterized by higher levels of LDL, TG, and CHOL and extremely high LDL levels compared to other clusters. Cluster B, encompassing 17,028 (22.7%) patients, was characterized by a younger age overall compared to other clusters and was labelled as Younger MASLD. Cluster C, comprising 12,103 patients (16.1%), was characterised by extremely high BMI and lower WHR than other clusters and labelled as Obesity MASLD. Cluster D, including 21,627 (28.8%) patients, exhibited the highest age and extremely high MLR compared to other clusters and was labelled as Inflammatory MASLD. Cluster E, consisting of 3678 (4.9%) patients, demonstrated elevated ALT and AST levels compared to other clusters and was labelled as Hepatotoxic MASLD. The results of the training cohort were consistent with those of the validation and internal test cohorts from UK Biobank as well as external test cohort from Nanfang Hospital (shown in Supplemental Tables 3–5; Supplemental Figs. 4–6).

**Table 2:** Baseline characteristic of training cohort

Characteristic	Dyslipidemia n = 20,682^1^	Younger n = 17,028^1^	Obesity n = 12,103^1^	Inflammatory n = 21,627^1^	Hepatotoxic n = 3678^1^	*P*^2^	Effect size^3^
Age	60 (55, 64)	48 (44, 51)	57 (51, 62)	63 (59, 66)	57 (51, 62)	< 0.001	0.50423122
Sex	11,384 (55%)	13,822 (81%)	1067 (8.8%)	18,306 (85%)	2613 (71%)	< 0.001	0.31981731
Race						< 0.001	0.05723753
White	19,314 (93%)	15,064 (88%)	11,040 (91%)	20,200 (93%)	3359 (91%)		0.05723753
Else	1368 (6.6%)	1964 (12%)	1063 (8.8%)	1427 (6.6%)	319 (8.7%)		0.05723753
BMI	29.5 (27.7, 31.7)	29.8 (28.0, 31.9)	35.9 (33.3, 39.2)	30.0 (28.1, 32.2)	30.8 (28.3, 33.7)	< 0.001	0.10101695
WHR	0.92 (0.88, 0.96)	0.94 (0.91, 0.98)	0.85 (0.81, 0.89)	0.97 (0.93, 1.00)	0.95 (0.91, 0.99)	< 0.001	0.23996071
ALT	25 (20, 33)	29 (22, 38)	21 (17, 28)	25 (20, 33)	61 (47, 79)	< 0.001	0.20563056
AST	26 (23, 31)	27 (23, 31)	23 (20, 27)	26 (23, 31)	53 (48, 63)	< 0.001	0.17666491
ALB	45.50 (43.90, 47.16)	45.90 (44.25, 47.54)	43.89 (42.28, 45.53)	44.90 (43.25, 46.55)	45.73 (43.92, 47.53)	< 0.001	0.09305343
TG	2.43 (1.85, 3.23)	2.25 (1.65, 3.08)	1.75 (1.32, 2.33)	2.01 (1.51, 2.73)	2.17 (1.56, 3.06)	< 0.001	0.01318530
CHOL	7.05 (6.61, 7.60)	5.78 (5.27, 6.22)	5.60 (5.03, 6.11)	5.07 (4.49, 5.59)	5.84 (5.12, 6.50)	< 0.001	0.48100451
HDL-C	1.33 (1.17, 1.53)	1.15 (1.01, 1.32)	1.31 (1.14, 1.52)	1.16 (1.01, 1.36)	1.23 (1.05, 1.46)	< 0.001	0.25152048
LDL-C	4.65 (4.32, 5.06)	3.72 (3.34, 4.06)	3.53 (3.09, 3.93)	3.15 (2.70, 3.55)	3.69 (3.15, 4.22)	< 0.001	0.45513641
HbA1c	5.45 (5.24, 5.66)	5.32 (5.10, 5.54)	5.48 (5.25, 5.73)	5.47 (5.23, 5.74)	5.48 (5.22, 5.78)	< 0.001	0.08573332
MLR	0.23 (0.18, 0.29)	0.24 (0.19, 0.30)	0.21 (0.16, 0.26)	0.29 (0.23, 0.37)	0.26 (0.20, 0.33)	< 0.001	0.07440376

^1^Values are median (IQR) or n (%); ^2^*P* value: Pearson's Chi-squared test; Kruskal-Wallis rank sum test; ^3^Effect size for ANOVA

*BMI* body mass index, *WHR* waist-hip ratio, *ALT* alanine aminotransferase, *AST* aspartate aminotransferase, *TG* triglyceride, *CHOL* cholesterol, *HDL-C* high-density lipoprotein cholesterol, *LDL-C* low-density lipoprotein cholesterol, *HbA1c* Hemoglobin A1c, *MLR* monocyte/lymphocyte ratio

### Comparisons of risk of metabolic complications among the clusters

**Fig. 3:**
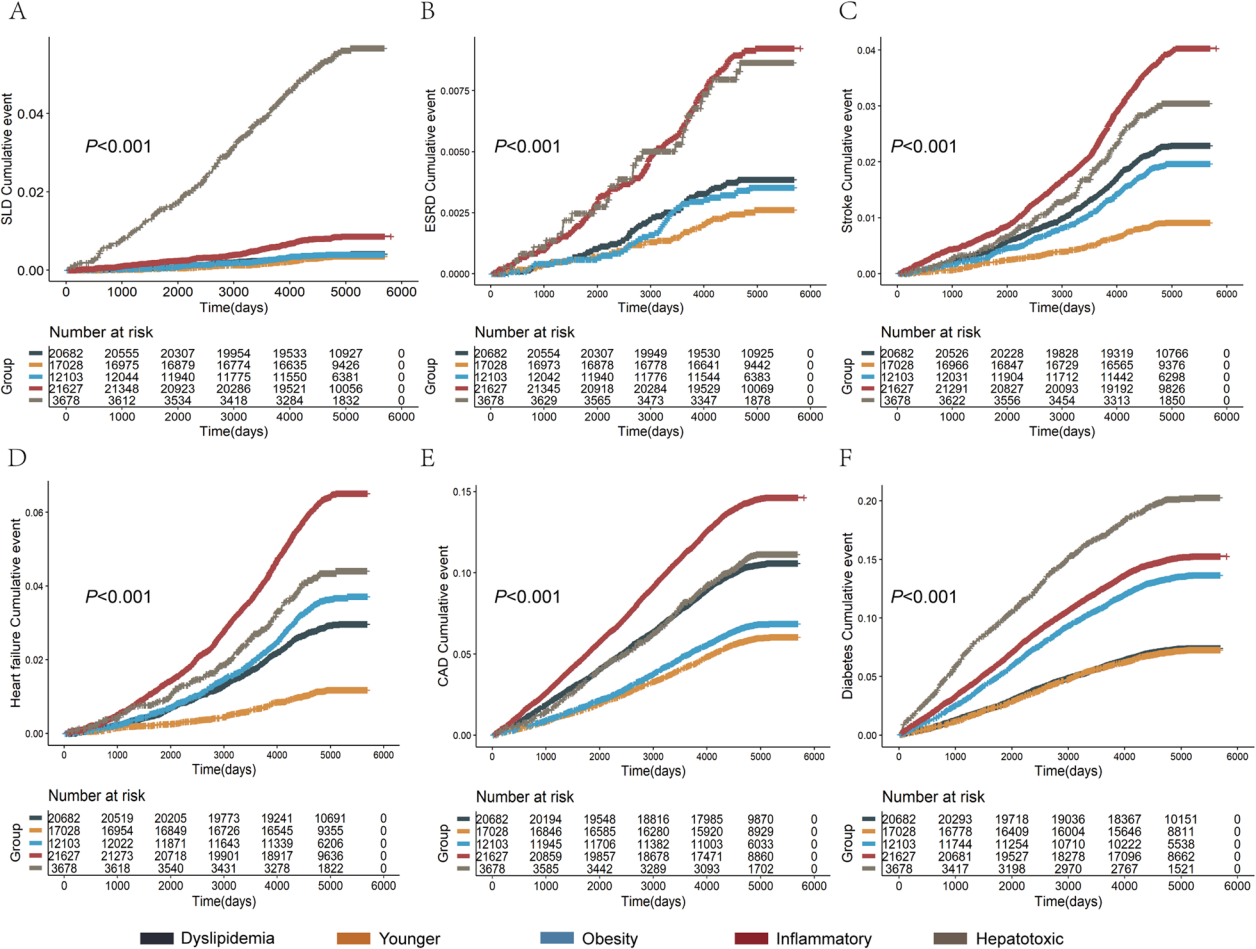
Cumulative incidence of different outcomes over time by clusters in the training cohort. Cumulative events of SLD (**A**), ESRD (**B**), stroke (**C**), heart failure (**D**), CAD (**E**) and diabetes (**F**) in different clusters in training cohort. SLD, severe liver diseases; ESRD, end-stage renal diseases; CAD, coronary artery diseases

We compared the risk of intrahepatic and extrahepatic complications (SLD, ESRD, stroke, HF, CAD, and diabetes) in each cluster (Fig. 3). We found that the highest risk of SLD occurrence in Hepatotoxic MASLD was more than 13 times that in Metabolic-Dyslipidemia MASLD (HR = 13.9, 95% CI 10.7–18.1; *P* < 0.001) in the training cohort with a median follow-up of 4989 days (Table 3). Inflammatory MASLD showed significant risk of SLD (HR = 1.99, 95% CI 1.52–2.61; *P* < 0.001); however, Younger MASLD and Obesity MASLD were not significantly different from Metabolic-Dyslipidemia MASLD. In the training cohort, the risk of ESRD in Inflammatory MASLD (HR = 2.13, 95% CI 1.62–2.80; *P* < 0.001) and Hepatotoxic MASLD (HR = 2.14, 95% CI 1.41–3.28; *P* < 0.001) was higher than that of Metabolic-Dyslipidemia MASLD. Inflammatory MASLD had the highest risk of stroke (HR = 1·69, 95% CI 1.50–1.91; *P* < 0.001), while Younger MASLD had the lowest risk (HR = 0.38, 95% CI 0.32–0.46; *P* < 0.001). Inflammatory MASLD also had the highest risk of HF (HR = 2.08, 95% CI 1.87–2.30; *P* < 0.001), followed by Hepatotoxic MASLD (HR = 1.47, 95% CI 1.23–1.76; *P* < 0.001), while Younger MASLD had the lowest risk (HR = 0.37, 95% CI 0.31–0.43; *P* < 0.001). Additionally, Inflammatory MASLD had the highest risk of CAD occurrence (HR = 1.21, 95% CI 1.14–1.28; *P* < 0·001), while Younger MASLD had the lowest risk (HR = 0·47, 95% CI 0.44–0.51; *P* < 0·001). Hepatotoxic MASLD had the highest risk of diabetes (HR = 3·08, 95% CI: 2·82–3·37; *P* < 0.001), followed by Inflammatory MASLD (HR = 2.16, 95% CI 2.03–2.31; *P* < 0.001) (Table 3). The results of the training cohort were consistent with those of the validation and test cohorts (Supplemental Figs. 7–8; Supplemental Table 6; Table 3).

**Table 3:** The associations between different clusters of training, validation and internal test cohort and clinical outcomes using multivariate Cox regression analysis

Cluster	Train		Validation		Test	
	HR (95% CI)	*P*	HR (95% CI)	P	HR (95% CI)	P
SLD						
Younger	0.83 (0.59, 1.16)	0.27	0.76 (0.40, 1.43)	0.39	1.38 (0.68, 2.84)	0.37
Obesity	1.17 (0.80, 1.71)	0.41	1.31(0.70,2.42)	0.40	2.77 (1.35, 5.68)	0.005
Inflammatory	1.99 (1.52, 2.61)	< 0.001	1.95 (1.20, 2.42)	0.007	4.17 (2.32, 7.63)	< 0.001
Hepatotoxic	13.9 (10.7, 18.1)	< 0.001	18.9 (12.0,29.7)	< 0.001	27.2(15.3, 48.60)	< 0.001
ESRD						
Younger	0.59 (0.40, 0.86)	0.006	0.83 (0.44,1.58)	0.006	0.50 (0.23, 1.07)	0.07
Obesity	1.02 (0.68, 1.53)	0.91	1.14 (0.57, 2.28)	0.70	1.40 (0.72, 2.71)	0.31
Inflammatory	2.13 (1.62, 2.80)	< 0.001	3.24 (1.99, 5.27)	< 0.001	2.71 (1.64, 4.47)	< 0.001
Hepatotoxic	2.14 (1.41, 3.28)	< 0.001	2.07 (0.85, 2.07)	0.20	3.13 (1.54, 6.35)	0.001
Stroke						
Younger	0.38 (0.32, 0.46)	< 0.001	0.38 (0.27, 0.52)	< 0.001	0.46 (0.35, 0.63)	< 0.001
Obesity	0.86 (0.73, 1.03)	0.09	0.82 (0.61, 1.09)	0.17	0.72 (0.53, 0.97)	0.03
Inflammatory	1.69 (1.50, 1.91)	< 0.001	1.50 (1.22, 1.86)	< 0.001	1.70 (1.38, 2.09)	< 0.001
Hepatotoxic	1.33 (1.08, 1.65)	0.008	1.35 (0.93, 1.95)	0.11	1.60 (1.13, 2.25)	0.007
Heart failure						
Younger	0.37 (0.31, 0.43)	< 0.001	0.37 (0.28, 0.48)	< 0.001	0.37 (0.28, 0.49)	< 0.001
Obesity	1.35 (1.18, 1.55)	< 0.001	1.27 (1.01, 1.60)	0.04	1.18 (0.94, 1.49)	0.14
Inflammatory	2.08 (1.87, 2.30)	< 0.001	1.80 (1.51, 2.13)	< 0.001	1.75 (1.47, 2.09)	< 0.001
Hepatotoxic	1.47 (1.23, 1.76)	< 0.001	1.43 (1.06, 1.94)	0.02	1.53 (1.13, 2.06)	0.005
CAD						
Younger	0.47 (0.44, 0.51)	< 0.001	0.41 (0.35, 0.47)	< 0.001	0.45 (0.39, 0.51)	< 0.001
Obesity	0.79(0.73, 0.86)	< 0.001	0.82 (0.70, 0.95)	0.009	0.78 (0.67, 0.90)	< 0.001
Inflammatory	1.21 (1.14, 1.28)	< 0.001	1.13 (1.02, 1.25)	0.01	1.16 (1.05, 1.29)	0.000
Hepatotoxic	0.97 (0.87, 1.09)	0.11	0.91 (0.75, 1.10)	0.35	0.94 (0.78, 1.14)	0.56
Diabetes						
Younger	0.95 (0.88, 1.03)	0.24	0.89 (0.78, 1.03)	0.11	0.96 (0.83, 1.09)	0.47
Obesity	1.66 (1.54, 1.79)	< 0.001	1.46 (1.28, 1.66)	< 0.001	1.73 (1.52, 1.97)	< 0.001
Inflammatory	2.16 (2.03, 2.31)	< 0.001	2.02 (1.81, 2.25)	< 0.001	2.35 (2.10, 2.63)	< 0.001
Hepatotoxic	3.08 (2.82, 3.37)	< 0.001	2.67 (2.27, 3.13)	< 0.001	3.30 (2.83, 3.86)	< 0.001

The hazard ratios were obtained with Dyslipidemia as reference group. Adjusted for sex, smoking status and alcohol intake. SLD, severe liver diseases; ESRD, end-stage renal diseases; CAD, coronary artery diseases

### GWAS analysis and pathway

Patients with subtypes Inflammatory MASLD and Hepatotoxic MASLD had a significantly increased risk of severe complications (SLD, ESRD, stroke, and HF). Therefore, we combined these subtypes in subsequent analyses and labeled them as high-risk group and the remaining three groups were combined and labeled as low-risk group. Supplemental Fig. 9 shows that high-risk group had a higher rate of serious complications than the low-risk group (*P* < 0.001). To explore the genetic association of SNPs with different prognosis in MASLD, we analyzed autosomal SNPs and identified 970 conditionally independent signals associated with high-risk group mapping to loci at *P* < 5 × 10^−8^. Supplemental Fig. 10 A, B shows that differential SNPs were mainly concentrated on chromosomes 1 and 19. Supplemental Fig. 10 C shows an SNP density diagram. The top 50 SNPs by* P* value were shown in Supplemental Table 7 and Supplemental Fig. 11. The risks of SNPs, like rs58542926 and rs738409 variants, were higher in the high-risk group than in the low-risk group in Supplemental Table 7 and Supplemental Fig. 11 (High risk vs. Low risk, OR [95%CI] 1.33 [1.28,1.38] and 1.19 [1.16,1.22]). Gene Ontology (GO) analysis of differentially expressed genes (DEGs) identified a striking enrichment of biological functions related to lipid homeostasis (Supplemental Fig. 12). In molecular function, DEGs were significantly enriched in LDL particle receptor binding binding (P = 3.2 × 10^−5^) and lipoprotein particle receptor binding (P = 5.9 × 10^−5^), while biological process analysis highlighted triglyceride metabolism (adjusted P = 2.6 × 10^−7^) and plasma lipoprotein particle clearance (P = 2.9 × 10^−7^) as dominant pathays. Cellular component analysis further revealed plasma lipoprotein particle (P = 2.65 × 10^−6^) and lipoprotein particle (P = 2.66 × 10^−6^), underscoring the role of subcellular structures in lipid processing. These findings, while descriptive, reveal a robust correlation between genetic variation and lipid-related biological processes.

## Discussion

In the large and most representative study of machine learning-informed subtype definition and risk prediction in MASLD, we firstly identified 5 main clusters with rigorous internal validation and external test using a clustering algorithm. Second, we highlighted high complications in individuals with MASLD and demonstrated important differences across subtypes. Two clusters with a higher risk of intrahepatic and extrahepatic complications were identified as high-risk group, and the other 3 subtypes as low-risk group. Of greatest interest was the differential SNPs between the high- and low-risk group that were mainly concentrated on chromosomes 1 and 19, of which the annotated genes enriched pathways were primarily related to lipid metabolism and transport.

Risk stratification and management are pertinent for patients with steatotic liver diseases; however, there is substantial scope for improvement for clinical, public health and research applications in primary and secondary prevention, including precision medicine. Romeo S’ team generated two partitioned polygenic risk scores based on the presence of lipoprotein retention in the liver, which suggested the presence of at least two distinct types of MASLD, one confined to the liver resulting in a more aggressive liver disease and one that is systemic and results in a higher risk of cardiometabolic disease [33]. Pattou et al. identified two distinct endotypes of at-risk MASLD, namely, cardiometabolic MASLD and live-specific MASLD based on six clinical factors; one showed rapid progression of chronic liver disease but limited risk of cardiovascular disease, and the other cluster, was primarily associated with dysglycemia and high levels of triglycerides, leading to a similar incidence of chronic liver disease but a higher risk of cardiovascular disease and type 2 diabetes [34]. However, other metabolic outcomes like renal diseases were not included in their study. Previous studies also have established Genetic Risk Score models based on MASLD-related SNPs, which had an increased risk of cirrhosis and liver cancer [35]. Patients with muscle-reducing MASLD are at an increased risk of liver fibrosis and coronary diseases [36, 37]. However, the feasibility and applicability of these methods are not robust. Ye et al. categorized MAFLD using demographic data and metabolic indicators to predict the risk of extrahepatic complications; however, no further investigations have been conducted regarding intrahepatic complications [38]. In our study, a comprehensive assessment of both intrahepatic and extrahepatic complications was conducted to provide a valuable framework for developing personalized treatments for individuals with MASLD. Our study also found that elevated inflammatory status seemed linked to worse outcomes in MASLD individuals. As is known, MASLD can further progress to inflammatory conditions such as metabolic-associated steatohepatitis (MASH), which subsequently lead to hepatic fibrosis, cirrhosis, and eventually hepatocellular carcinoma (HCC) [22]. This intricate process involves the concerted action of liver-resident cells. These highly plastic immune cells exhibit remarkable phenotypic flexibility, adapting to their specific spatial and temporal pathophysiological environment [39, 40]. Consequently, they emerge as a central hub connecting tissue organization, inflammation, and fibrosis. Their function and polarization are tightly regulated by metabolic dysregulation, highlighting their critical role in MASLD progression [41].

To improve risk stratification in MASLD, we explored the genetic validation of high- and low-risk groups, focusing on established variants linked to progressive outcomes. The PNPLA3 rs738409 variant (BETA = 0.17, *P* = 1.97 × 10^−43^), is a known modulator of hepatic lipid droplet homeostasis [42]. A common non-synonymous polymorphism in TM6SF2 rs58542926 was recently associated with increased hepatic triglyceride content, but also promotes clinically relevant hepatic fibrosis [43, 44]. In addition, cholesterol homeostasis is closely related to metabolic-related diseases [45]. Several studies have revealed that the inducer of LDL receptor degraders ubiquitinates low-density lipoprotein receptor (LDLR) intracellularly, thus marking its degradation. At the same time, the proprotein convertase subtilisin-kexin type 9 (PCSK9) regulates LDLR proteolysis through intracellular or extracellular binding to the receptor [44]. Extracellular binding of circulating PCSK9 to LDLR at the plasma membrane is followed by endocytosis of the PCSK9–LDLR complex [46]. Moreover, RNA-targeted therapy blocking the PCSK9-mediated LDLR degradation pathway successfully reduces plasma LDL cholesterol and the risk of atherosclerotic cardiovascular disease [47].

This study has some unsolved limitations. First, diagnostic alternatives we used were based on non-invasive calculations (FLI score and FIB-4 score) to define hepatic steatosis and fibrosis in UK Biobank, which may need to be validated by further studies using imaging data or liver tissue biopsy pathology. Nevertheless, the feasibility of imaging and biopsy in large population is relatively limited. Second, we identified five subtypes via unsupervised K-means clustering method in a European cohort as the training set and in a Chinese cohort as the external test set, which needs to be further validated in larger or other regions populations. We hoped to collect detailed follow-up information from the Nanfang Hospital cohort and validate our results in UK Biobank further. In addition, a key limitation of this GWAS is its inherent inability to prove causation. Across molecular function, biological process, and cellular component analyses, differentially expressed genes consistently clustered into lipid metabolism-related pathways. This cross-category consistency implies a non-random association with lipid homeostasis, though the underlying mechanisms remain to be elucidated. Finally, whether the different subtypes can be transformed from one subtype into another is unclear.

## Conclusion

Our study improves the ability to identify the risk of intra- and extra-hepatic complications in patients with MASLD by developing a new classification system based on seven clinical characteristics. Classification of patients into five categories may help personalize treatment to reduce the risk of complications associated with MASLD.

## Supplementary Information

Supplementary Material 1

## Data Availability

Researchers can request the UK Biobank data we used at the website (www.ukbiobank.ac.uk/). Data of the Health examinee dataset in Nanfang Hospital is available from the corresponding author upon reasonable request.
