# Supplementary material for: Novel subtypes of metabolic associated steatotic liver disease linked to clinical outcomes: implications for precision medicine
Source: J Transl Med. 2025 Jul 10;23:769. doi: 10.1186/s12967-025-06670-5 (PMC12247398; doi:10.1186/s12967-025-06670-5)
Supplement: Supplementary file 1 — Supplementary Material 1 [file 12967_2025_6670_MOESM1_ESM.pdf]

Supplemental Materials

**Supplemental Method 1.** Inclusion and exclusion criteria in UK Biobank.	**Page 2**
**Supplemental Table 1.** Fields' ID and corresponding field used in the analysis..	**Page 3**
**Supplemental Table 2.** Baseline characteristic of training, validation and internal test cohort from UK Biobank.	**Page 5**
**Supplemental Table 3.** Baseline characteristic of validation cohort.	**Page 7**
**Supplemental Table 4.** Baseline characteristic of internal test cohort.	**Page 9**
**Supplemental Table 5.** Baseline characteristic of external test cohort.	**Page 11**
**Supplemental Table 6.** The associations between different clusters of training, validation and internal test cohort and clinical outcomes using univariate Cox regression analysis.	**Page 13**
**Supplemental Table 7.** Summary of top 50 SNP variations in the MASLD high-risk/low-risk analysis.	**Page 16**
**Supplemental Fig. 1.** Workflow of study design.	**Page 20**
**Supplemental Fig. 2.** The correlation between the selected variables and FIB-4 scores.	**Page 21**
**Supplemental Fig. 3.** Cluster characteristics of patients of baseline in training cohort.	**Page 22**
**Supplemental Fig. 4.** Cluster characteristics of patients of baseline in validation cohort.	**Page 23**
**Supplemental Fig. 5.** Cluster characteristics of patients of baseline in internal test cohort.	**Page 24**
**Supplemental Fig. 6.** Cluster characteristics of patients of baseline in external test cohort.	**Page 25**
**Supplemental Fig. 7.** Prognosis of outcomes over time by clusters in the validation cohort.	**Page 26**
**Supplemental Fig. 8.** Prognosis of outcomes over time by clusters in the internal test cohort.	**Page 27**
**Supplemental Fig. 9.** Cumulative events of severe complications in high-risk and low-risk group.	**Page 28**
**Supplemental Fig. 10.** Manhattan plot, Q-Q plot, density plot of SNP.	**Page 29**
**Supplemental Fig.11.** Forest plot of Significant SNP (Top 50 by *P* value).	**Page 30**
**Supplemental Fig. 12.** Results of GO analysis.	**Page 31**

Supplemental Method 1. Inclusion and exclusion criteria in UK Biobank.

In UK Biobank, we further excluded individuals who: 1) had a history of diabetes at baseline (n=14,282); 2) had a history of stroke at baseline (n=2,189); 3) had coronary artery disease (CAD) at baseline (n=22,355); 4) had a history of heart failure (HF) at baseline (n=2,866); 5) were diagnosed with severe liver disease (SLD) before the baseline survey using the International Classification of Diseases, 10th Edition (ICD-10, I85.0) (n=523); and 6) were diagnosed with end-stage renal disease (ESRD) before baseline assessment either through self-reporting or health system registries (n=506). Thereafter, we excluded participants who lacked baseline requirements (n=48,113). Following this, patients lacking baseline indicators or having extreme laboratory indicators that could affect statistical analysis were excluded. This study exclusively included individuals diagnosed with MASLD and excluded non-MASLD populations (n=221,832). In this study, the lowest 1% and highest 1% were extremes; therefore, 2% of values were removed (n=29,160). The final analysis included 125,197 participants with complete serological indicators, age, sex, and other characteristics (shown in Figure 1).

Supplemental Table 1. Fields' ID and corresponding field used in the analysis.

FieldID	Field
31	Sex
48	Waist circumference
49	Hip circumference
21000	Ethnic background
21001	Body mass index (BMI)
21003	Age when attended assessment centre
30120	Lymphocyte count
30130	Monocyte count
30620	Alanine aminotransferase
30650	Aspartate aminotransferase
30690	Cholesterol
30730	Gamma glutamyltransferase
30750	Glycated haemoglobin (HbA1c)
30760	HDL cholesterol
30780	LDL direct
30870	Triglycerides
40000	Date of death
40005	Date of cancer diagnosis
40006	Type of cancer: ICD10
40008	Age at cancer diagnosis
40009	Reported occurrences of cancer
40011	Histology of cancer tumour

40012	Behaviour of cancer tumour
40013	Type of cancer: ICD9
41202	Diagnoses - main ICD10
41203	Diagnoses - main ICD9
41204	Diagnoses - secondary ICD10
41205	Diagnoses - secondary ICD9
41210	Operative procedures - secondary OPCS4
41270	Diagnoses - ICD10
41271	Diagnoses - ICD9
41272	Operative procedures - OPCS4
41273	Operative procedures - OPCS3

Supplemental Table 2. Baseline characteristic of training, validation and internal test cohort from UK Biobank.

Characteristic	**Training** n = 75,118	**Validation** n = 25,039	**Internal Test** n = 25,040	***P* value**
Sex	47,192 (63%)	15,808 (63%)	15,791 (63%)	0.6
Age	58 (51, 63)	58 (51, 63)	58 (51, 63)	0.5
Race				0.7
White	68,977 (92%)	22,950 (92%)	22,977 (92%)	
Else	6,141 (8.2%)	2,090 (8.3%)	2,062 (8.2%)	
BMI	30.5 (28.3, 33.3)	30.4 (28.2, 33.3)	30.4 (28.2, 33.3)	0.01
WHR	0.93 (0.88, 0.98)	0.93 (0.88, 0.98)	0.93 (0.88, 0.98)	0.8
ALT	26 (20, 35)	26 (20, 35)	26 (20, 35)	0.5
AST	26 (22, 31)	26 (22, 31)	26 (22, 31)	0.99
ALB	45.18 (43.47, 46.90)	45.17 (43.46, 46.87)	45.20 (43.46, 46.93)	0.3
TG	2.14 (1.58, 2.91)	2.15 (1.59, 2.92)	2.14 (1.59, 2.92)	0.4
CHOL	5.86 (5.11, 6.63)	5.84 (5.11, 6.62)	5.85 (5.11, 6.61)	0.8
HDL-C	1.23 (1.07, 1.44)	1.23 (1.07, 1.43)	1.24 (1.07, 1.44)	0.6

Characteristic	**Training**<br>n = 75,118	**Validation**<br>n = 25,039	**Internal Test**<br>n = 25,040	***P* value**
LDL-C	3.76 (3.18, 4.34)	3.75 (3.17, 4.33)	3.75 (3.17, 4.33)	0.5
HbA1c	5.43 (5.20, 5.67)	5.43 (5.21, 5.66)	5.43 (5.20, 5.67)	0.2
MLR	0.25 (0.19, 0.31)	0.25 (0.19, 0.31)	0.25 (0.19, 0.31)	0.9

Note: values are median (IQR) or n (%); *P* value: Pearson's Chi-squared test; Kruskal-Wallis rank sum test.

Supplemental Table 3. Baseline characteristic of validation cohort.

Characteristic	Dyslipidemia N = 6,880[1]	Younger N = 5,690[1]	Obesity N = 4,008[1]	Inflammatory N = 7,244[1]	Hepatotoxic N = 1,217[1]	*P*[2]	Effect size[3]
Age	60 (55, 64)	48 (44, 51)	57 (51, 62)	63 (59, 66)	56 (50, 62)	<0.001	0.50829699
Sex	3,825 (56%)	4,665 (82%)	344 (8.6%)	6,063 (84%)	894 (73%)	<0.001	0.32393808
Race						<0.001	0.06455717
White	6,408 (93%)	5,007 (88%)	3,698 (92%)	6,763 (93%)	1,101 (90%)		0.06455717
Else	472 (6.9%)	683 (12%)	310 (7.7%)	481 (6.6%)	116 (9.5%)		0.06455717
BMI	29.5 (27.6, 31.6)	29.6 (27.8, 31.7)	35.9 (33.3, 39.3)	29.9 (28.0, 32.2)	30.8 (28.4, 33.5)	<0.001	0.11028771
WHR	0.92 (0.88, 0.96)	0.94 (0.91, 0.97)	0.85 (0.82, 0.89)	0.97 (0.93, 1.00)	0.95 (0.91, 0.99)	<0.001	0.24283306
ALT	25 (20, 33)	29 (22, 38)	21 (17, 28)	25 (20, 33)	62 (47, 78)	<0.001	0.20760348
AST	26 (23, 31)	27 (23, 31)	23 (20, 27)	27 (23, 31)	53 (47, 62)	<0.001	0.17046397
ALB	45.48 (43.86, 47.20)	45.94 (44.28, 47.58)	43.91 (42.24, 45.53)	44.93 (43.29, 46.62)	45.77 (43.69, 47.54)	<0.001	0.09745983
TG	2.44 (1.88, 3.25)	2.24 (1.64, 3.08)	1.76 (1.33, 2.33)	2.02 (1.51, 2.73)	2.21 (1.59, 3.26)	<0.001	0.01293270
CHOL	7.05 (6.60, 7.59)	5.76 (5.25, 6.21)	5.60 (5.03, 6.12)	5.08 (4.50, 5.60)	5.82 (5.14, 6.48)	<0.001	0.48672908

Characteristic	Dyslipidemia N = 6,880[1]	Younger N = 5,690[1]	Obesity N = 4,008[1]	Inflammatory N = 7,244[1]	Hepatotoxic N = 1,217[1]	***P***[2]	Effect size[3]
HDL-C	1.33 (1.17, 1.53)	1.15 (1.01, 1.32)	1.32 (1.14, 1.52)	1.16 (1.01, 1.36)	1.22 (1.04, 1.44)	<0.001	0.25423337
LDL-C	4.64 (4.31, 5.04)	3.70 (3.31, 4.04)	3.51 (3.07, 3.93)	3.16 (2.71, 3.56)	3.69 (3.19, 4.20)	<0.001	0.46096818
HbA1c	5.45 (5.23, 5.66)	5.32 (5.11, 5.54)	5.49 (5.25, 5.73)	5.46 (5.23, 5.73)	5.47 (5.22, 5.77)	<0.001	0.08375070
MLR	0.23 (0.18, 0.30)	0.24 (0.19, 0.29)	0.21 (0.17, 0.26)	0.29 (0.23, 0.37)	0.27 (0.21, 0.33)	<0.001	0.07139580

Note: values are median (IQR) or n (%); *P* value: Pearson's Chi-squared test; Kruskal-Wallis rank sum test; [3] Effect size for ANOVA (small ⩾0.1, medium ⩾0.25, large ⩾0.4).

BMI, body mass index; WHR, waist-hip ratio; ALT, alanine aminotransferase; AST, aspartate aminotransferase; TG, triglyceride; CHOL, cholesterol; HDL-C, high-density lipoprotein cholesterol; LDL-C, low-density lipoprotein cholesterol; HbA1c, Hemoglobin A1c; MLR, monocyte/lymphocyte ratio.

Supplemental Table 4. Baseline characteristic of internal test cohort

Characteristic	Dyslipidemia N = 6,854[1]	Younger N = 5,734[1]	Obesity N = 3,975[1]	Inflammatory N = 7,271[1]	Hepatotoxic N = 1,206[1]	***P***[2]	Effect size[3]
Age	60 (55, 63)	48 (44, 51)	57 (51, 62)	63 (59, 66)	57 (51, 62)	<0.001	0.495075977
Sex	3,794 (55%)	4,643 (81%)	376 (9.5%)	6,140 (84%)	855 (71%)	<0.001	0.313902450
Race						<0.001	0.059307371
White	6,377 (93%)	5,049 (88%)	3,642 (92%)	6,780 (93%)	1,102 (91%)		0.059307371
Else	477 (7.0%)	685 (12%)	333 (8.4%)	491 (6.8%)	104 (8.6%)		0.059307371
BMI	29.5 (27.7, 31.7)	29.7 (27.9, 31.9)	35.9 (33.3, 39.2)	29.9 (28.0, 32.2)	30.8 (28.4, 33.8)	<0.001	0.104511498
WHR	0.92 (0.88, 0.96)	0.94 (0.91, 0.97)	0.85 (0.81, 0.89)	0.97 (0.93, 1.00)	0.95 (0.91, 0.99)	<0.001	0.233736559
ALT	26 (20, 33)	29 (22, 38)	21 (17, 28)	26 (20, 33)	63 (48, 78)	<0.001	0.203789083
AST	26 (23, 31)	27 (23, 31)	23 (20, 27)	27 (23, 31)	53 (47, 62)	<0.001	0.171989283
ALB	45.50 (43.87, 47.16)	45.85 (44.23, 47.43)	43.88 (42.34, 45.54)	44.86 (43.23, 46.58)	45.63 (43.79, 47.41)	<0.001	0.088082423
TG	2.43 (1.86, 3.23)	2.27 (1.66, 3.10)	1.73 (1.32, 2.32)	2.03 (1.52, 2.72)	2.13 (1.54, 2.95)	<0.001	0.008864767
CHOL	7.05 (6.62, 7.60)	5.76 (5.27, 6.21)	5.61 (5.04, 6.11)	5.06 (4.49, 5.60)	5.80 (5.16, 6.47)	<0.001	0.483453146

Characteristic	Dyslipidemia<br>N = 6,854[1]	Younger<br>N = 5,734[1]	Obesity<br>N = 3,975[1]	Inflammatory<br>N = 7,271[1]	Hepatotoxic<br>N = 1,206[1]	***P***[2]	**Effect size**[3]
HDL-C	1.33 (1.17, 1.52)	1.15 (1.01, 1.32)	1.32 (1.15, 1.52)	1.16 (1.01, 1.35)	1.24 (1.07, 1.46)	<0.001	0.250366254
LDL	4.65 (4.32, 5.05)	3.71 (3.32, 4.05)	3.54 (3.09, 3.93)	3.14 (2.70, 3.55)	3.70 (3.19, 4.18)	<0.001	0.461940232
HbA1c	5.45 (5.24, 5.65)	5.32 (5.10, 5.54)	5.47 (5.26, 5.72)	5.47 (5.23, 5.74)	5.45 (5.20, 5.76)	<0.001	0.084341287
MLR	0.23 (0.18, 0.29)	0.24 (0.19, 0.29)	0.21 (0.17, 0.26)	0.29 (0.23, 0.37)	0.26 (0.20, 0.33)	<0.001	0.058829498

Note: values are median (IQR) or n (%); *P* value: Pearson's Chi-squared test; Kruskal-Wallis rank sum test; [3] Effect size for ANOVA (small ⩾0.1, medium ⩾0.25, large ⩾0.4).

BMI, body mass index; WHR, waist-hip ratio; ALT, alanine aminotransferase; AST, aspartate aminotransferase; TG, triglyceride; CHOL, cholesterol; HDL-C, high-density lipoprotein cholesterol; LDL-C, low-density lipoprotein cholesterol; HbA1c, Hemoglobin A1c; MLR, monocyte/lymphocyte ratio.

Supplemental Table 5. Baseline characteristic of external test cohort.

Characteristic	Dyslipidemia N = 298[1]	Younger N = 285[1]	Obesity N = 107[1]	Inflammatory N = 267[1]	Hepatotoxic N = 38[1]	P^2	Cohen's f^3
Age	49 (45, 54)	36 (32, 39)	45 (39, 49)	51 (47, 57)	44 (37, 50)	<0.001	0.741142055
Sex	178 (60%)	186 (65%)	49 (46%)	189 (71%)	25 (66%)	<0.001	
BMI	24.86 (23.26, 26.51)	25.27 (23.40, 26.83)	27.81 (25.76, 30.42)	25.29 (23.77, 26.90)	27.29 (23.88, 28.79)	<0.001	0.036843900
WHR	0.88 (0.84, 0.93)	0.89 (0.84, 0.93)	0.82 (0.77, 0.88)	0.91 (0.86, 0.97)	0.91 (0.87, 1.00)	<0.001	0.033988212
ALT	21(16, 30)	24(16, 30)	19(13, 27)	22(16, 30)	82(69, 118)	<0.001	0.121854556
AST	20 (17, 23)	20 (16, 24)	18 (15, 20)	20 (17, 23)	49 (44, 62)	<0.001	0.097588635
TG	1.87 (1.37, 2.59)	1.56 (1.17, 2.27)	1.42 (1.07, 2.20)	1.60 (1.12, 2.32)	1.75 (1.20, 2.66)	0.040	0.061419896
CHOL	6.47 (6.04, 6.96)	5.20 (4.71, 5.61)	4.99 (4.43, 5.57)	4.77 (4.29, 5.28)	5.26 (4.85, 5.85)	<0.001	0.420193352
HDL-C	1.39 (1.23, 1.56)	1.20 (1.05, 1.33)	1.22 (1.06, 1.40)	1.20 (1.05, 1.33)	1.20 (1.06, 1.40)	<0.001	0.230379108
LDL-C	4.15 (3.83, 4.52)	3.29 (2.92, 3.56)	3.13 (2.70, 3.44)	2.97 (2.48, 3.25)	3.33 (2.97, 3.67)	<0.001	0.365617323
Glucose	5.07 (4.72, 5.61)	4.83 (4.55, 5.14)	4.95 (4.66, 5.30)	5.14 (4.79, 5.58)	5.22 (4.61, 5.64)	<0.001	0.182423702
MLR	0.17 (0.14, 0.21)	0.17 (0.14, 0.20)	0.16 (0.13, 0.20)	0.21 (0.17, 0.26)	0.19 (0.16, 0.25)	<0.001	0.073386422

Note: values are median (IQR) or n (%); *P* value: Pearson's Chi-squared test; Kruskal-Wallis rank sum test; ;[3] Effect size for ANOVA (small ⩾0.1, medium ⩾0.25,

large ≥0.4)

BMI, body mass index; WHR, waist-hip ratio; ALT, alanine aminotransferase; AST, aspartate aminotransferase; TG, triglyceride; CHOL, cholesterol; HDL-C, high-density lipoprotein cholesterol; LDL-C, low-density lipoprotein cholesterol; MLR, monocyte/lymphocyte ratio.

Supplemental Table 6. The associations between different clusters of training, validation and internal test cohort and clinical outcomes using univariate Cox regression analysis.

**Cluster**	**Train**		**Validation**		**Internal Test**	
	HR (95% CI)	*P*	HR (95% CI)	P	HR (95% CI)	P
**SLD**						
Younger	0.86 (0.62, 1.21)	0.39	0.76 (0.40, 1.41)	0.38	1.41 (0.70, 2.87)	0.33
Obesity	0.99 (0.70, 1.43)	0.99	1.30 (0.71, 2.34)	0.40	2.33 (1.17, 4.64)	0.02
Inflammatory	2.13 (1.63, 2.78)	<0.001	1.94(1.20, 3.14)	0.006	4.38 (2.46, 7.82)	<0.001
Hepatotoxic	14.4 (11.1, 18.6)	<0.001	18.7(12.0, 29.4)	<0.001	27.8 (15.6, 49.5)	<0.001
**ESRD**						
Younger	0.66 (0.45, 0.96)	0.03	0.91 (0.48, 1.27)	0.78	0.53 (0.25, 1.12)	0.09
Obesity	0.90 (0.62, 1.32)	0.60	1.16 (0.60, 2.24)	0.66	1.41 (0.75, 2.62)	0.28
Inflammatory	2.39 (1.83, 3.11)	<0.001	3.50 (2.18, 5.61)	<0.001	2.86 (1.77, 4.65)	<0.001
Hepatotoxic	2.24 (1.47, 3.41)	<0.001	2.10 (0.94, 4.73)	0.07	3.15 (1.56, 6.38)	0.001
**Stroke**						
Younger	0.40 (0.33, 0.47)	<0.001	0.38 (0.27, 0.52)	<0.001	0.47 (0.35, 0.64)	<0.001

Obesity	0.86 (0.73, 1.00)	0.05	0.84 (0.63, 1.10)	0.21	0.73 (0.55, 0.98)	0.03
Inflammatory	1.74 (1.56, 1.96)	<0.001	1.51 (1.23, 1.85)	<0.001	1.72 (1.41, 2.10)	<0.001
Hepatotoxic	1.34 (1.08, 1.65)	0.007	1.33 (0.93, 1.93)	0.12	1.58 (1.13, 2.23)	0.008
**Heart failure**						
Younger	0.39 (0.33, 0.46)	<0.001	0.40 (0.30, 0.52)	<0.001	0.38 (0.29, 0.50)	<0.001
Obesity	1.25 (1.01, 1.41)	<0.001	1.19 (0.96, 1.47)	0.09	1.18 (0.95, 1.46)	0.13
Inflammatory	2.23 (2.02, 2.46)	<0.001	1.93 (1.63, 2.28)	<0.001	1.81 (1.53, 2.14)	<0.001
Hepatotoxic	1.51 (1.26, 1.80)	<0.001	1.48 (1.09, 2.00)	0.012	1.52 (1.13, 2.05)	0.006
**CAD**						
Younger	0.55 (0.51, 0.59)	<0.001	0.48 (0.42, 0.55)	<0.001	0.52 (0.46, 0.59)	<0.001
Obesity	0.63 (0.58, 0.68)	<0.001	0.62 (0.54, 0.72)	<0.001	0.65 (0.56, 0.75)	<0.001
Inflammatory	1.42 (1.34, 1.50)	<0.001	1.33 (1.21, 1.47)	<0.001	1.36 (1.23, 1.49)	<0.001
Hepatotoxic	1.05 (0.94, 1.17)	0.39	1.01 (0.83, 1.21)	0.95	0.99 (0.82, 1.21)	0.97
**Diabetes**						
Younger	0.98 (0.91, 1.05)	0.54	0.87 (0.76, 0.99)	0.04	0.97 (0.85, 1.12)	0.72
Obesity	1.90 (1.77, 2.04)	<0.001	1.78 (1.57, 2.01)	<0.001	1.99 (1.76, 2.25)	<0.001

Inflammatory	2.16 (2.02, 2.30)	<0.001	1.92 (1.73, 2.14)	<0.001	2.36 (2.12, 2.63)	<0.001
Hepatotoxic	3.00 (2.75, 3.28)	<0.001	2.51 (2.13, 3.94)	<0.001	3.16 (2.71, 3.70)	<0.001

Supplemental Table 7. Summary of top 50 SNP variations with significance in the MASLD high-risk/low-risk analysis.

CHR	SNP	BP	A1	Gene(nearest)	BETA	P	Function
19	rs1065853	109818306	T	APOE	0.441546	4.66E-124	
19	rs7412	109817192	T	APOE	0.440456	8.06E-124	lipid metabolism[1]
19	rs72654473	109818158	A	APOE	0.292561	2.28E-71	
19	rs190712692	109817838	A	APOE	0.398534	2.40E-63	
19	rs41290120	45411941	A	PVRL2	0.398467	5.83E-63	
19	rs141622900	45319631	A	APOE	0.393261	2.41E-62	
1	rs12740374	109816863	T	CELSR2	0.198912	2.87E-58	
1	rs646776	19393890	T	PSRC1	-0.19848	3.03E-58	lipid metabolism[2]
1	rs629301	19379549	T	CELSR2	-0.19837	3.31E-58	
1	rs7528419	19388500	G	CELSR2	0.19679	3.35E-57	lipid metabolism[3]
1	rs3832016	19407718	CT	CELSR2	-0.19631	1.32E-55	
1	rs660240	19370340	C	CELSR2	-0.19624	1.50E-55	
19	rs429358	45415713	C	APOE	-0.2214	2.18E-53	lipid metabolism[1]
19	rs118147862	45415935	A	BCAM	0.383839	4.50E-49	
1	rs552693039	45410002	TC	CELSR2	0.190071	6.00E-46	

19	rs200210321	44324727	AG	SUGP1	0.289555	6.57E-46	
19	rs58542926	45422160	T	TM6SF2	0.283066	2.31E-45	lipid metabolism[4]; liver fibrosis[5]
19	rs8107974	44324730	T	SUGP1	0.28107	3.67E-45	
19	rs10401969	45392254	C	SUGP1	0.277069	3.70E-44	lipid metabolism[6]
19	rs756350040	44324855	T	HAPLN4	0.285776	4.60E-44	
19	rs10414043	19419071	A	APOE	-0.21765	1.37E-43	
19	rs7256200	45422946	T	APOE	-0.2173	1.69E-43	
19	rs769449	45422846	A	APOE	-0.21703	1.81E-43	
22	rs738409	19460541	G	PNPLA3	0.173056	1.97E-43	Lipid droplets[7]; antioxidant response[8,9]
19	rs12721051	19366632	G	APOC1	-0.18286	2.65E-43	
22	rs738408	19432290	T	PNPLA3	0.172698	3.07E-43	
19	rs6857	45424351	T	PVRL2	-0.19043	4.11E-43	
22	rs3747207	45397229	A	PNPLA3	0.173184	4.11E-43	
19	rs739846	45400747	A	SUGP1	0.274586	4.56E-43	

19	rs4420638	19578743	G	APOE	-0.18182	5.14E-43	
19	rs56131196	19456917	A	APOE	-0.18142	9.11E-43	
19	rs73001065	44340904	C	MAU2	0.281113	6.05E-42	lipid metabolism[4]
19	rs72999033	19494483	T	HAPLN4	0.294665	6.92E-42	
19	19:19432290_AG_A	109814880	A	MAU2	0.27963	7.16E-41	
19	rs814573	19610596	T	APOE	-0.18474	1.03E-40	
19	rs1160983	19477877	A	TOMM40	0.389882	1.32E-40	lipid metabolism[10]
19	rs61679753	45402262	A	TOMM40	0.38969	1.35E-40	
19	rs73002956	109815252	G	GATAD2A	0.250378	1.59E-39	
19	rs58489806	45424514	T	MAU2	0.245555	2.54E-39	
22	rs2294915	45389596	T	PNPLA3	0.160757	3.45E-39	
19	rs150268548	19662220	A	SUGP1	0.275596	4.19E-39	lipid metabolism[4]
1	rs4970834	19658472	T	CELSR2	0.173953	4.59E-39	
19	rs3794991	109818306	T	GATAD2A	0.247943	4.98E-39	
19	rs56255430	109817192	C	SUGP1	0.247544	9.87E-39	
19	rs111784051	109818158	G	TOMM40	0.379284	1.03E-37	

1	rs611917	109817838	G	CELSR2	0.142973	1.10E-37	
19	rs157592	45411941	C	APOE	-0.17684	1.89E-37	
19	rs7254892	45319631	A	PVRL2	0.365747	2.45E-37	
19	rs17216525	109816863	T	PBX4	0.246052	3.04E-37	lipid metabolism[4]
19	rs16996148	19393890	T	PBX4	0.242393	1.09E-36	lipid metabolism[4]

SNP, single nucleotide polymorphism; CHR, chromosome; A1, minor allele; A2, major allele; SE, standard error; Nearest gene, Annotated by 3DSNP v1.0 and plink (https://www.http://omic.tech/3dsnpv2/).

[1] Palmer ND, et al. Allele-specific variation at APOE increases nonalcoholic fatty liver disease and obesity but decreases risk of Alzheimer's disease and myocardial infarction. Hum Mol Genet. 2021 Jul 9;30(15):1443-1456. doi: 10.1093/hmg/ddab096. PMID: 33856023; PMCID: PMC8283205.

[2] Ronald J, et al. Analysis of recently identified dyslipidemia alleles reveals two loci that contribute to risk for carotid artery disease. Lipids Health Dis. 2009 Dec 1;8:52. PMID: 19951432.

[3] Møller PL, et al. Sortilin as a Biomarker for Cardiovascular Disease Revisited. Front Cardiovasc Med. 2021 Apr 16;8:652584. doi: 10.3389/fcvm.2021.652584. PMID: 33937362.

[4] Deng GX, et al. Association of the NCAN-TM6SF2-CILP2-PBX4-SUGP1-MAU2 SNPs and gene-gene and gene-environment interactions with serum lipid levels. Aging (Albany NY). 2020 Jun 22;12(12):11893-11913.

[5] Liu YL, et al. TM6SF2 rs58542926 influences hepatic fibrosis progression in patients with non-alcoholic fatty liver disease. Nat Commun. 2014 Jun 30;5:4309. doi: 10.1038/ncomms5309. .

[6] Sakuma I, et al. Liver lipid droplet cholesterol content is a key determinant of metabolic dysfunction-associated steatohepatitis. Proc Natl Acad Sci U S A. 2025 May 6;122(18).

[7] Armisen J, et al. AZD2693, a PNPLA3 antisense oligonucleotide, for the treatment of MASH in 148M homozygous participants: Two randomized phase I trials. J Hepatol. 2025 Jan 9:S0168-8278(25)00003-0.

[8] Chen VL, et al. Genetic risk accentuates dietary effects on hepatic steatosis, inflammation and fibrosis in a population-based cohort. J Hepatol. 2024 Sep;81(3):379-388.

[9] Caddeo A, et al. Precision medicine and nucleotide-based therapeutics to treat steatotic liver disease. Clin Mol Hepatol. 2025 Feb;31(Suppl):S76-S93. doi: 10.3350/cmh.2024.0438. Epub 2024 Aug 5. PMID: 39103998; PMCID: PMC11925435.

[10]Gao C, et al. Exome Sequencing Identifies Genetic Variants Associated with Circulating Lipid Levels in Mexican Americans: The Insulin Resistance Atherosclerosis Family Study (IRASFS). Sci Rep. 2018 Apr 4;8(1):5603. doi: 10.1038/s41598-018-23727-2. PMID: 29618726; PMCID: PMC5884862.

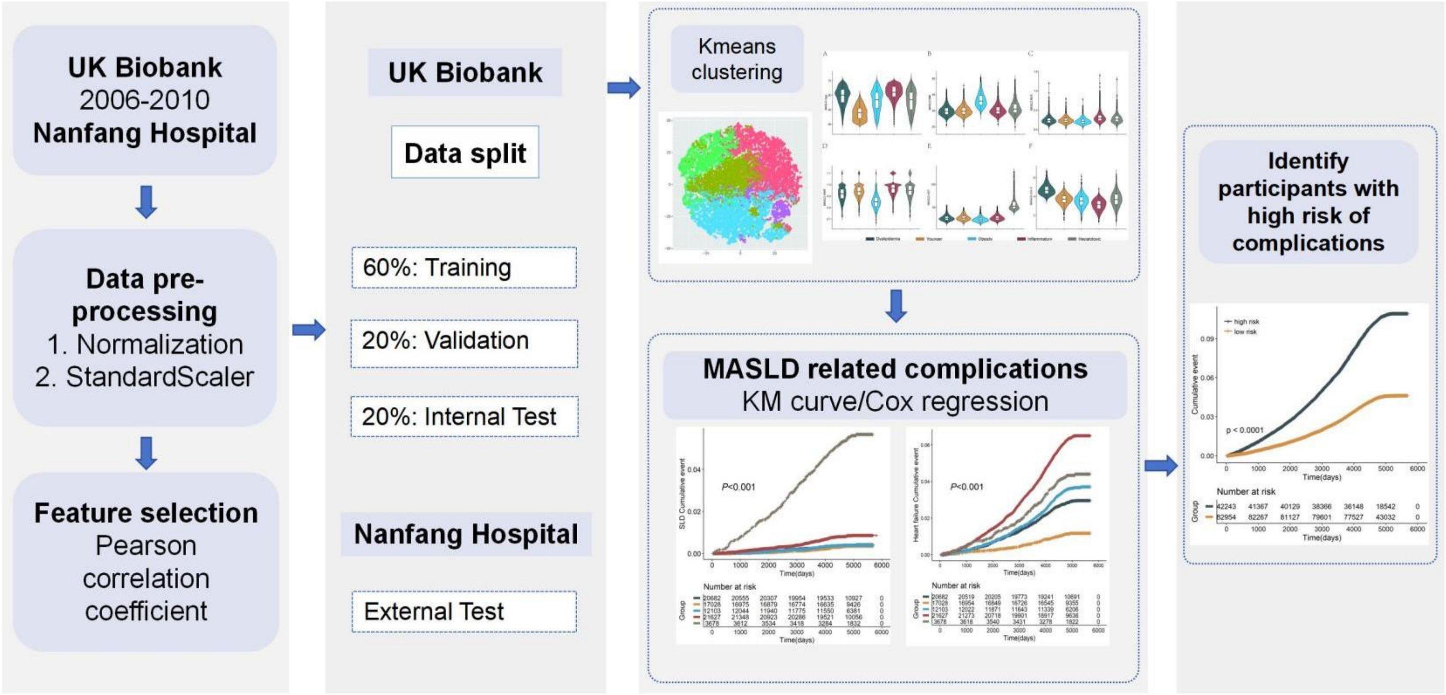

Supplemental Fig. 1. Workflow of study design.

A

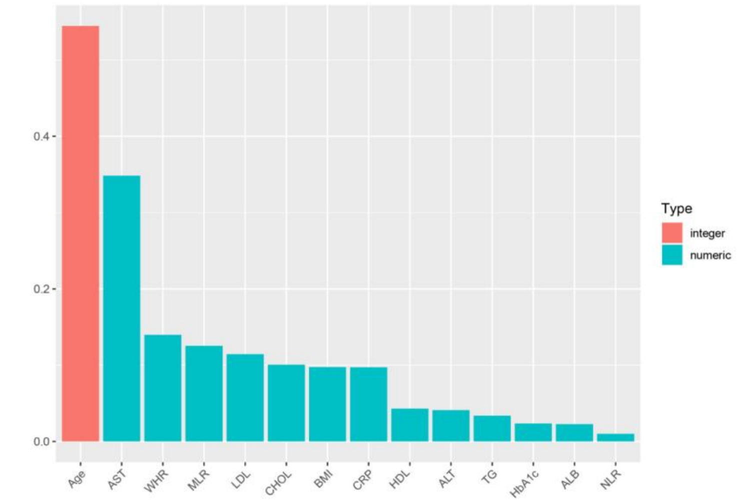

B

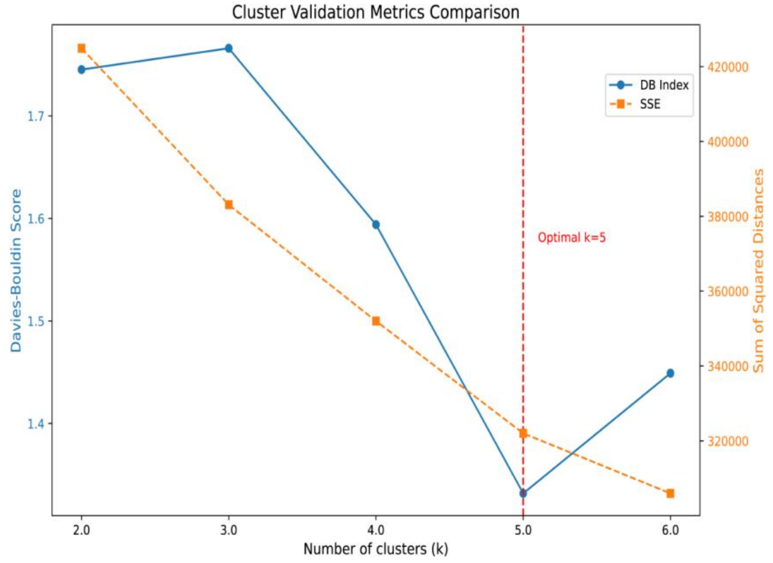

Supplemental Fig. 2. Selection of features and cluster number. (A)The correlation between the selected variables and FIB-4 scores. (B) Cluster Validation Metrics comparison.

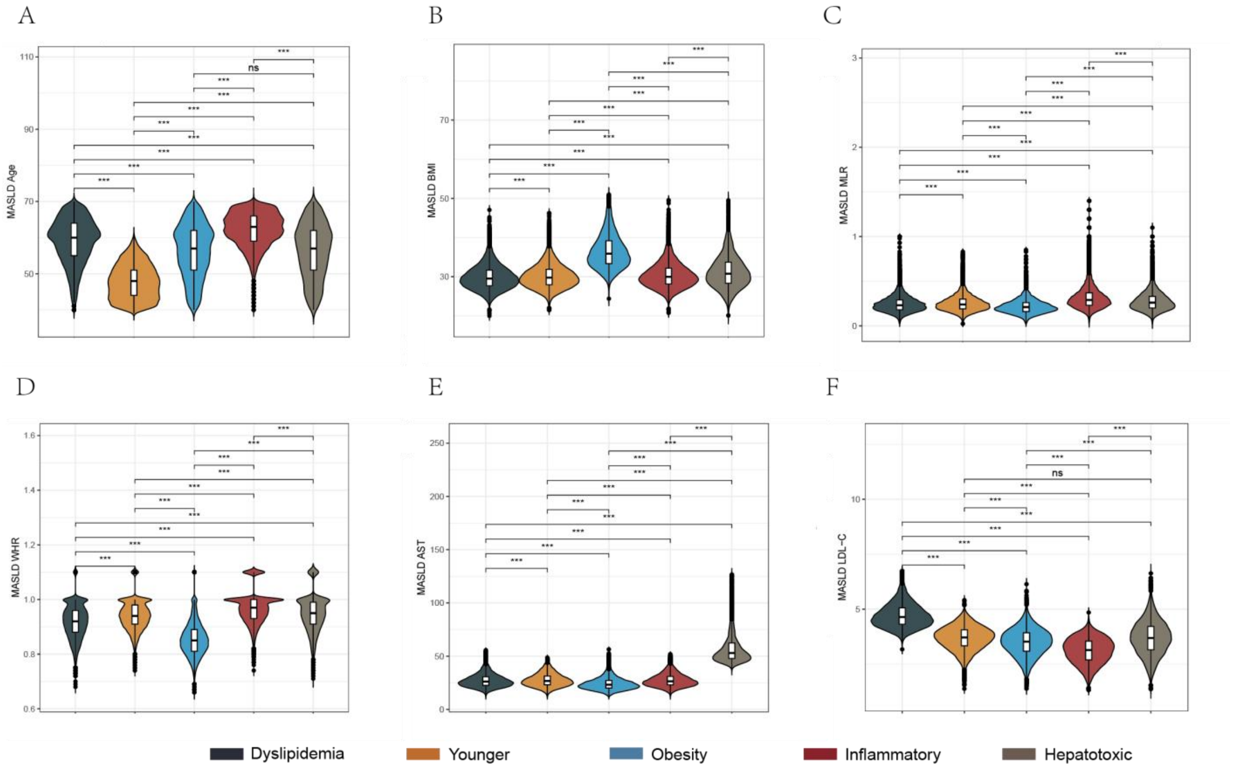

Supplemental Fig. 3. Cluster characteristics of patients of baseline in training cohort. Distributions of age (A), BMI (B), MLR (C), WHR (D), AST (E) and LDL-C (F) in different clusters.

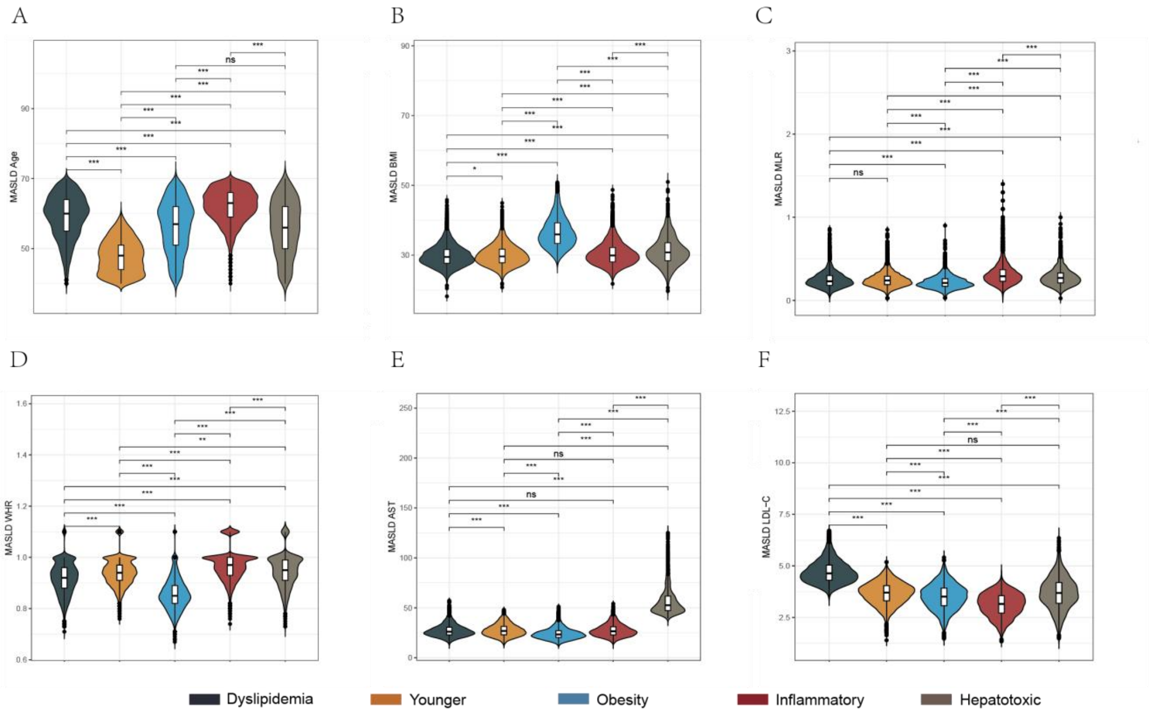

Supplemental Fig. 4. Cluster characteristics of patients of baseline in validation cohort. Distributions of age (A), BMI (B), MLR (C), WHR (D), AST (E) and LDL-C (F) in different clusters.

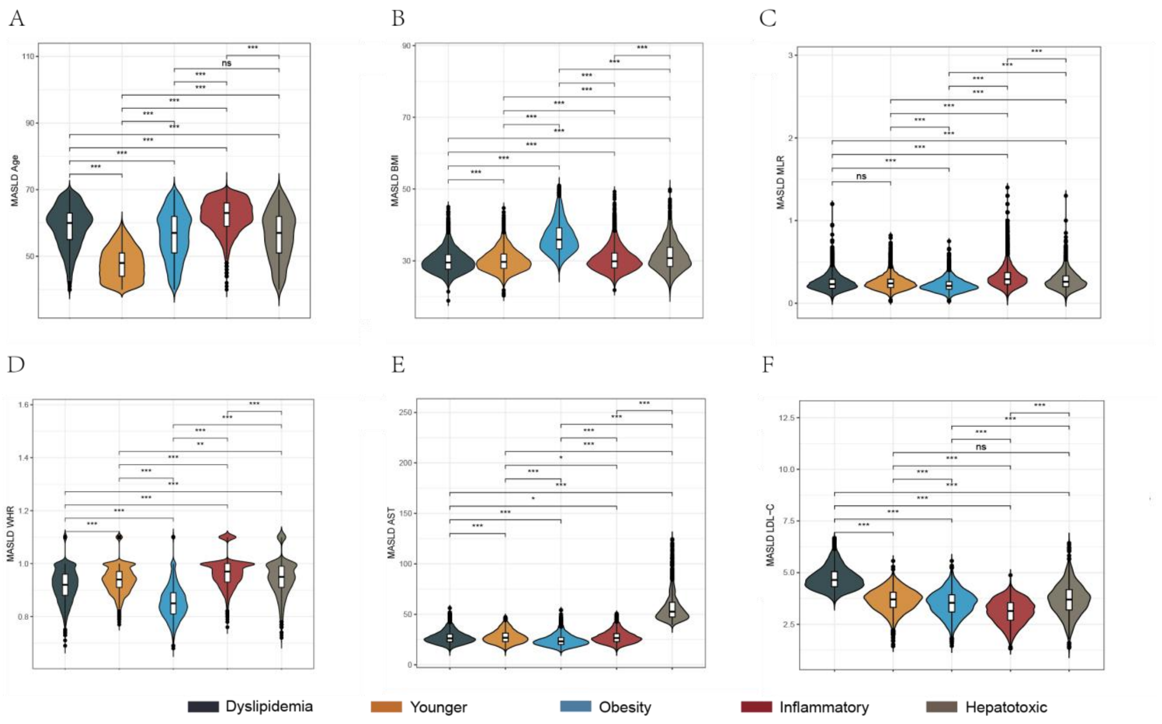

Supplemental Fig. 5. Cluster characteristics of patients of baseline in internal test cohort. Distributions of age (A), BMI (B), MLR (C), WHR (D), AST (E) and LDL-C (F) in different clusters.

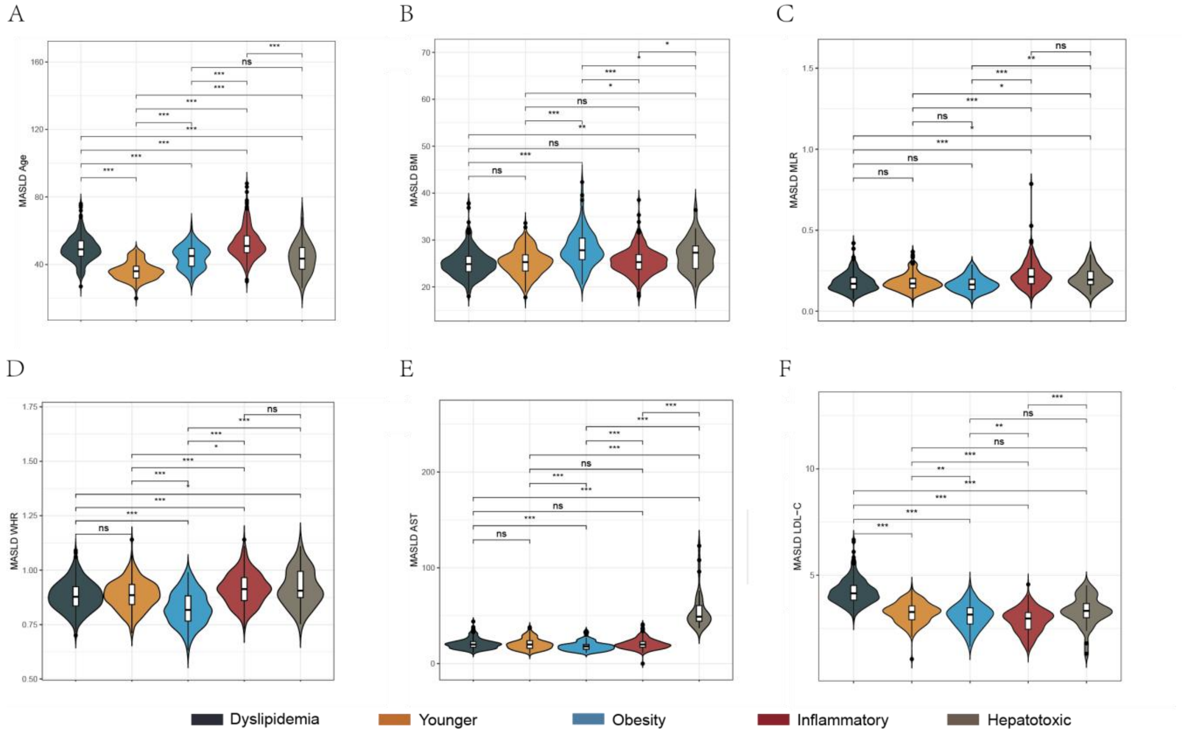

Supplemental Fig. 6. Cluster characteristics of patients of baseline in external test cohort. Distributions of age (A), BMI (B), MLR (C), WHR (D), AST (E) and LDL-C (F) in different clusters.

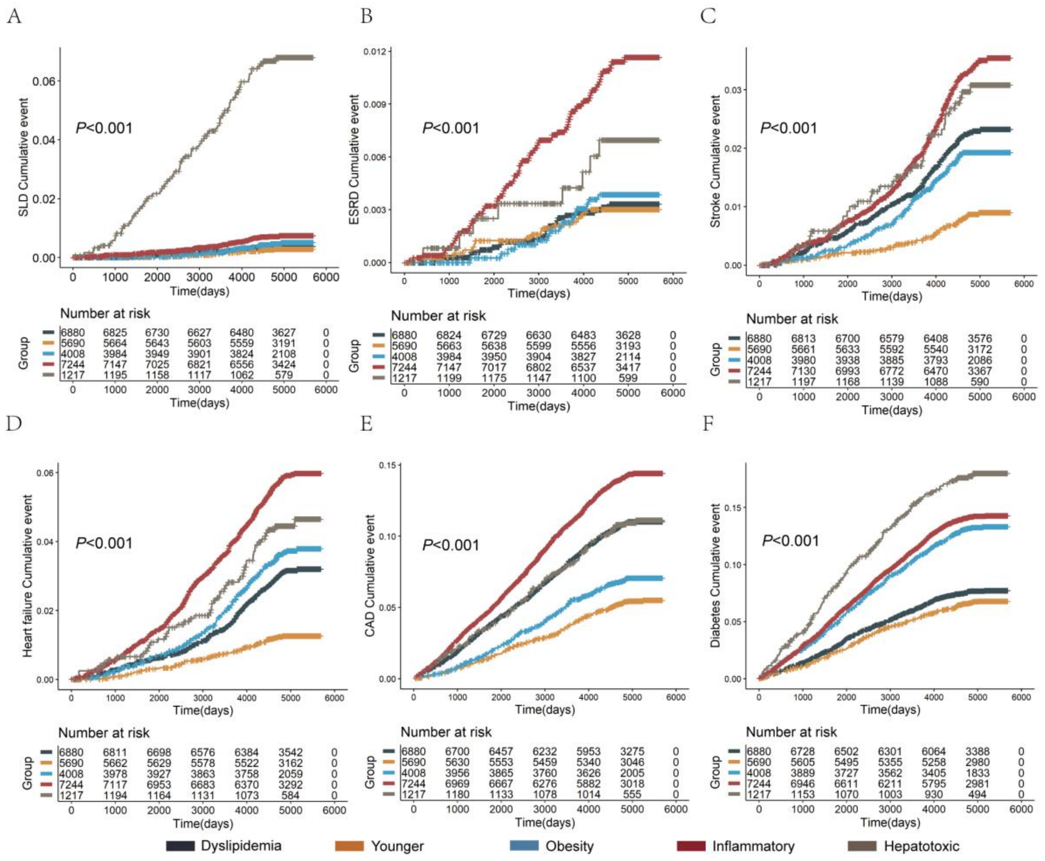

Supplemental Fig. 7. Prognosis of outcomes over time by clusters in the validation cohort. Cumulative events of SLD (A), ESRD (B), stroke (C), heart failure (D), CAD (E) and diabetes (F) in different clusters in validation cohort.

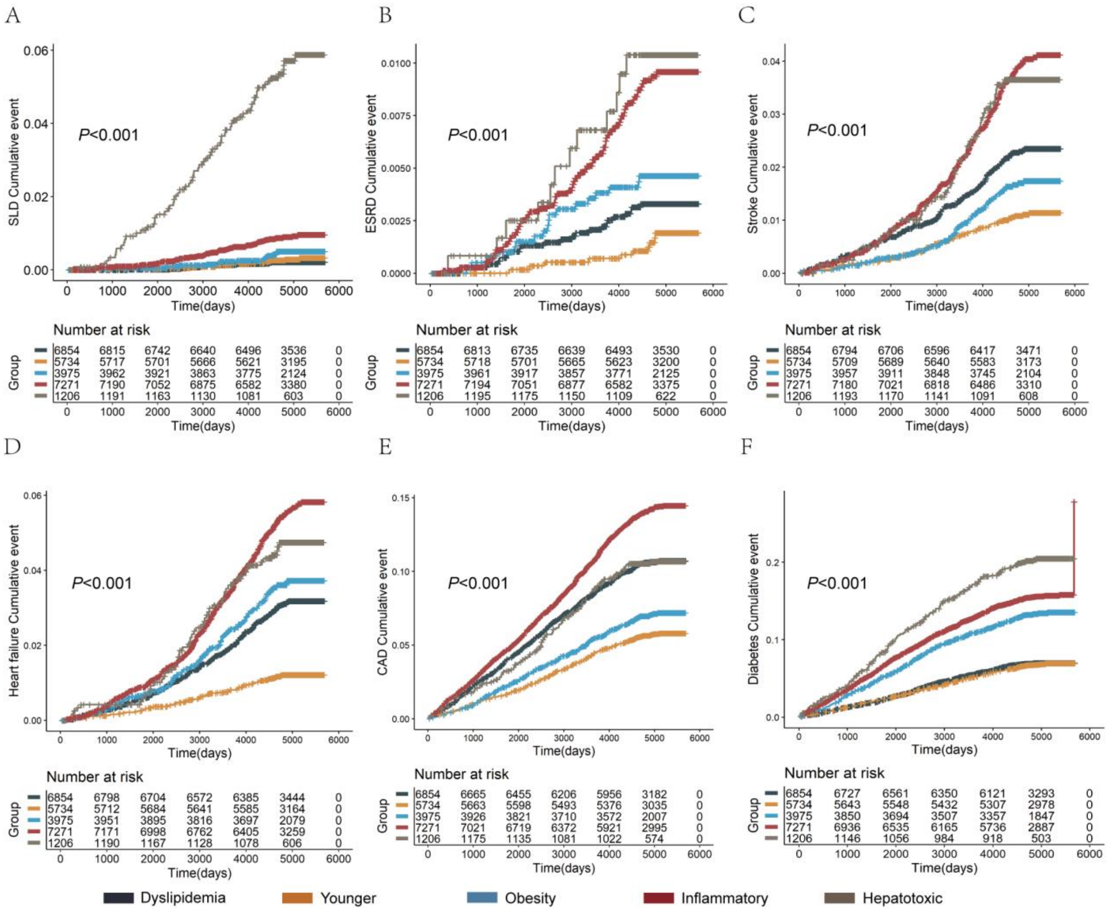

Supplemental Fig. 8. Prognosis of outcomes over time by clusters in the test cohort. Cumulative events of SLD (A), ESRD (B), stroke (C), heart failure (D), CAD (E) and diabetes (F) in different clusters in test cohort.

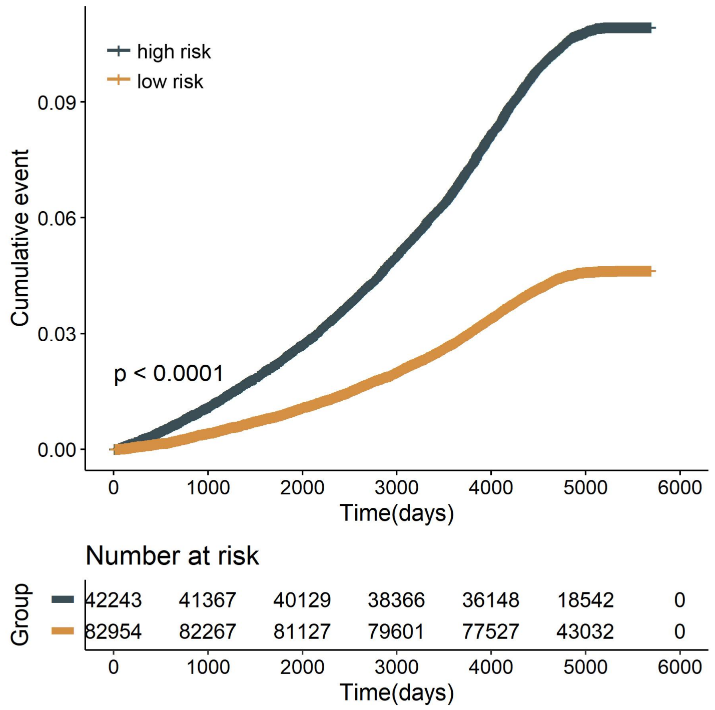

Supplemental Fig. 9. Cumulative events of severe complications in high-risk and low-risk groups.

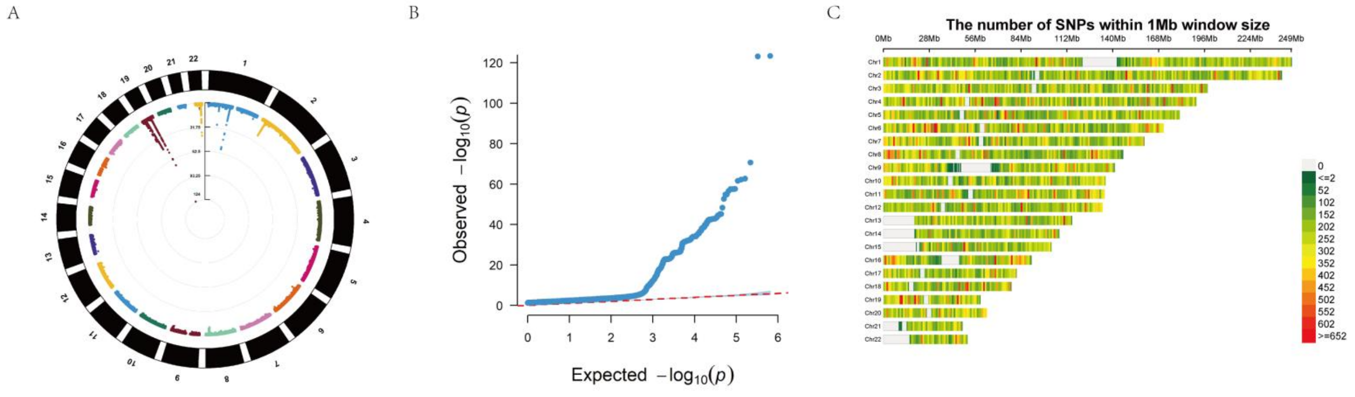

Supplemental Fig. 10. Manhattan plot (A), Q-Q plot (B), density plot (C) of SNP . (A)Differential SNPs were mainly concentrated on chromosomes 1 and 19 (with 5×10^{-8} as the threshold of *P* value). (B) Q-Q plot illustrated the relationship between observed (y - axis) and expected (x - axis) test statistics and are used as a tool for visualizing appropriate control of population substructure and the presence of association, which was based on a model adjusted for potential confounders, which brings the tail away from the y = x line. (C) Density plot known as the SNP density map, has different colors indicating the number of SNPS contained within 1Mb.

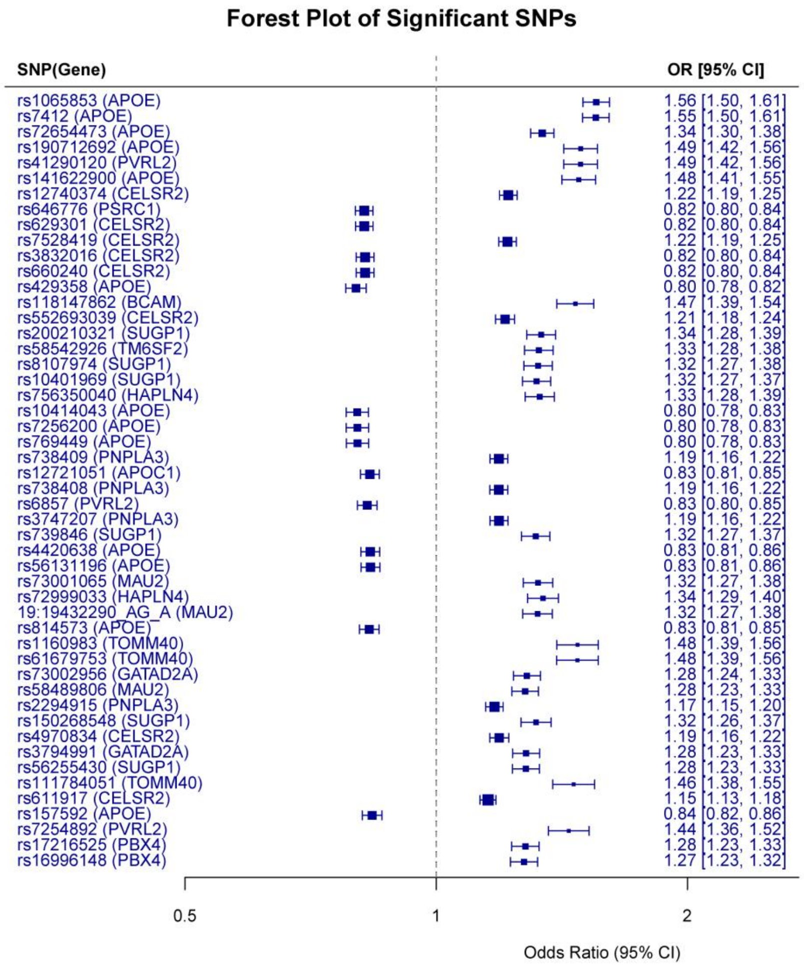

Supplemental Fig. 11. Forest plot of Significant SNP (Top 50 by *P* value).

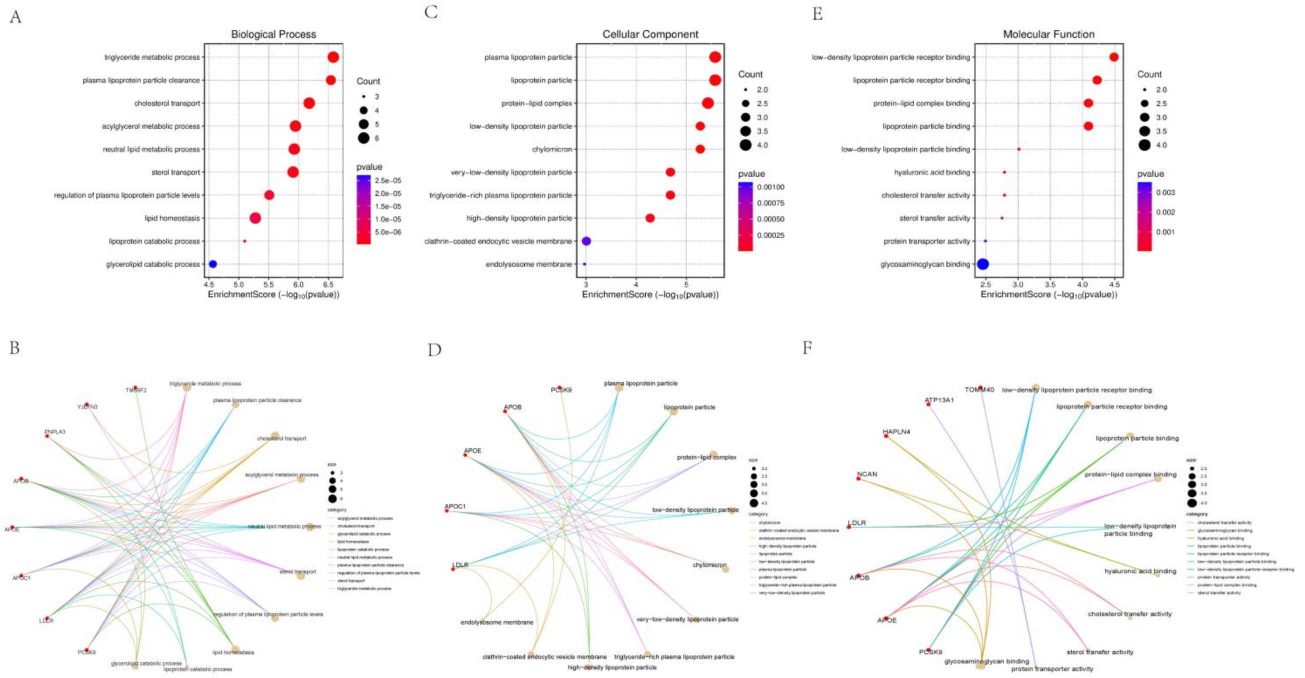

Supplemental Fig. 12. Results of GO analysis. The correlation between differential genes and biological functions enriched by gene ontology analysis. (F) Main enriched pathways for biological process(A-B), cell composition(C-D), and molecular function (E-F) based on gene ontology analysis.
